# Supplementary material for: Quantitative analysis of cis-regulatory elements in transcription with KAS-ATAC-seq
Source: Nat Commun. 2024 Aug 10;15:6852. doi: 10.1038/s41467-024-50680-8 (PMC11316786; doi:10.1038/s41467-024-50680-8)
Supplement: Supplementary file 1 [file 41467_2024_50680_MOESM1_ESM.pdf]

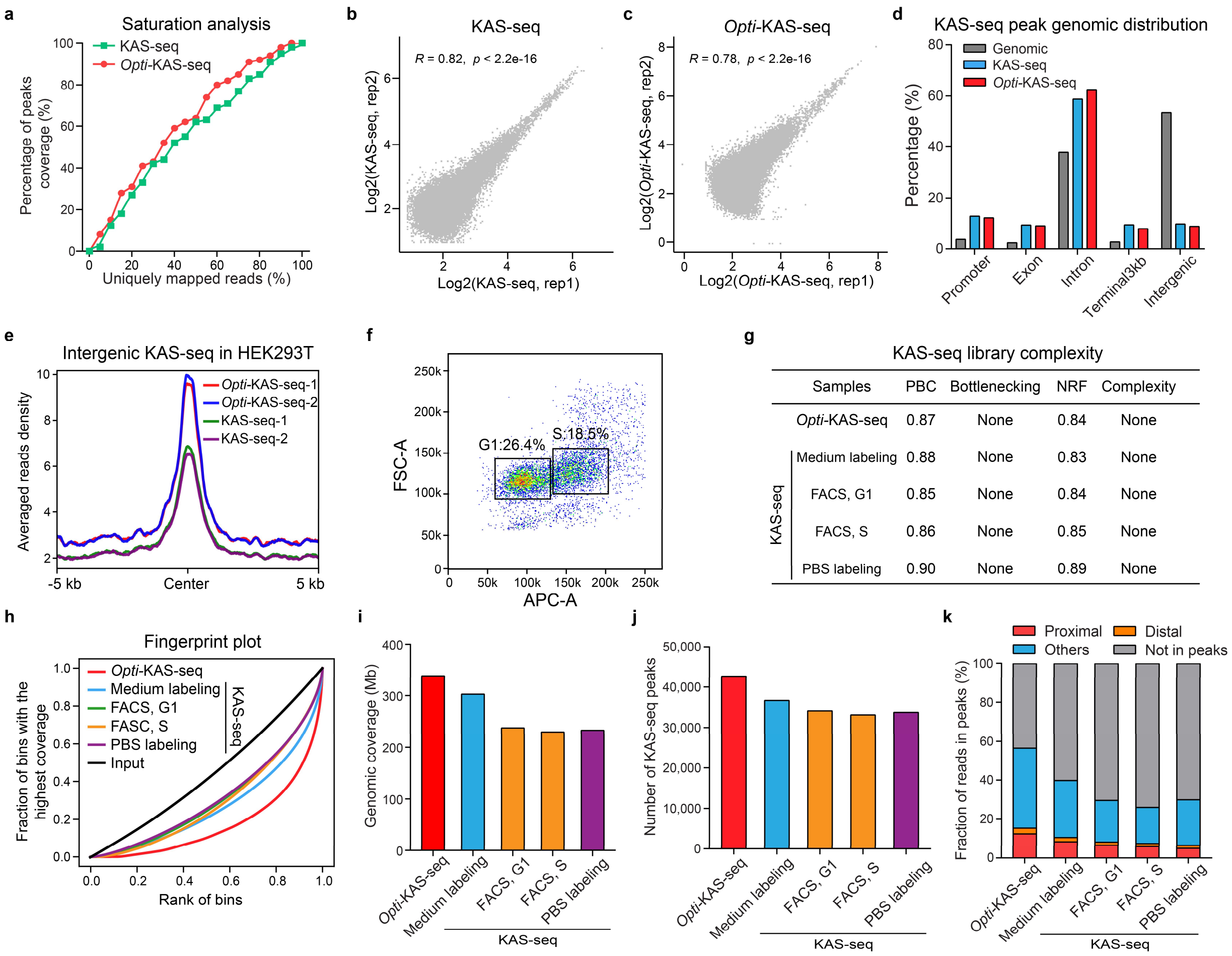

Supplementary Fig. 1: Comparing the quality control of KAS-seq and *Opti*-KAS-seq datasets in HEK293T cells.

a, Dotted line plot showing the saturation analysis of conventional KAS-seq and Optimized KAS-seq (*Opti*-KAS-seq) data in HEK293T cells. **b**,**c**, Scatterplots showing the Pearson correlation between two replicates of KAS-seq (**b**) and *Opti*-KAS-seq (**c**) data in HEK293T cells. Grey dots represent KAS-seq peaks (**b**, n = 54,073; **c**, n = 72,815). Both the Pearson correlation coefficients and significance (two-sided *p* values) are provided. **d**, Grouped bar plot illustrating the distribution of KAS-seq and *Opti*-KAS-seq peaks across various genomic features (promoter, exon, intron, terminator, and intergenic regions) in HEK293T cells. Promoters are defined as regions 2 kb upstream and downstream from the transcription start site (TSS). The relative proportions of each genomic feature, based on the hg19 Refseq annotation, are presented as 'Genomic'. **e**, Metagene profiles showing the averaged read density of *Opti*-KAS-seq and KAS-seq data across intergenic KAS-seq peaks (n = 2,446) in HEK293T cells. **f,** Gating strategy for HEK293T cell subsets in the G1 and S phases of the cell cycle. **g**, Table chart showing the library complexity metrics calculated using *Opti*-KAS-seq and KAS-seq data generated in HEK293T cells. **h**, Fingerprint plot of KAS-seq data from different protocols in HEK293T cells showing that the ssDNA capturing efficiency of *Opti*-KAS-seq is sig increased compared to KAS-seq. **i**, Vertical bar plot showing the genomic coverage of KAS-seq peaks from *Opti*-KAS-seq and KAS-seq data generated in HEK293T cells.

j, Vertical bar plot showing the number of KAS-seq peaks from *Opti*-KAS-seq and KAS-seq data generated in HEK293T cells. **k**, Stacked bar plot showing the fraction of reads in peaks (FRiP), calculated from KAS-seq and *Opti*-KAS-seq reads uniquely mapped to promoters (±500 bp of TSS), distal cis-regulatory elements (CREs) (>500 bp from TSS) and other regions in HEK293T cells. Each bar represents two merged technical replicates. All values were determined from 30 million randomly selected mapped reads.

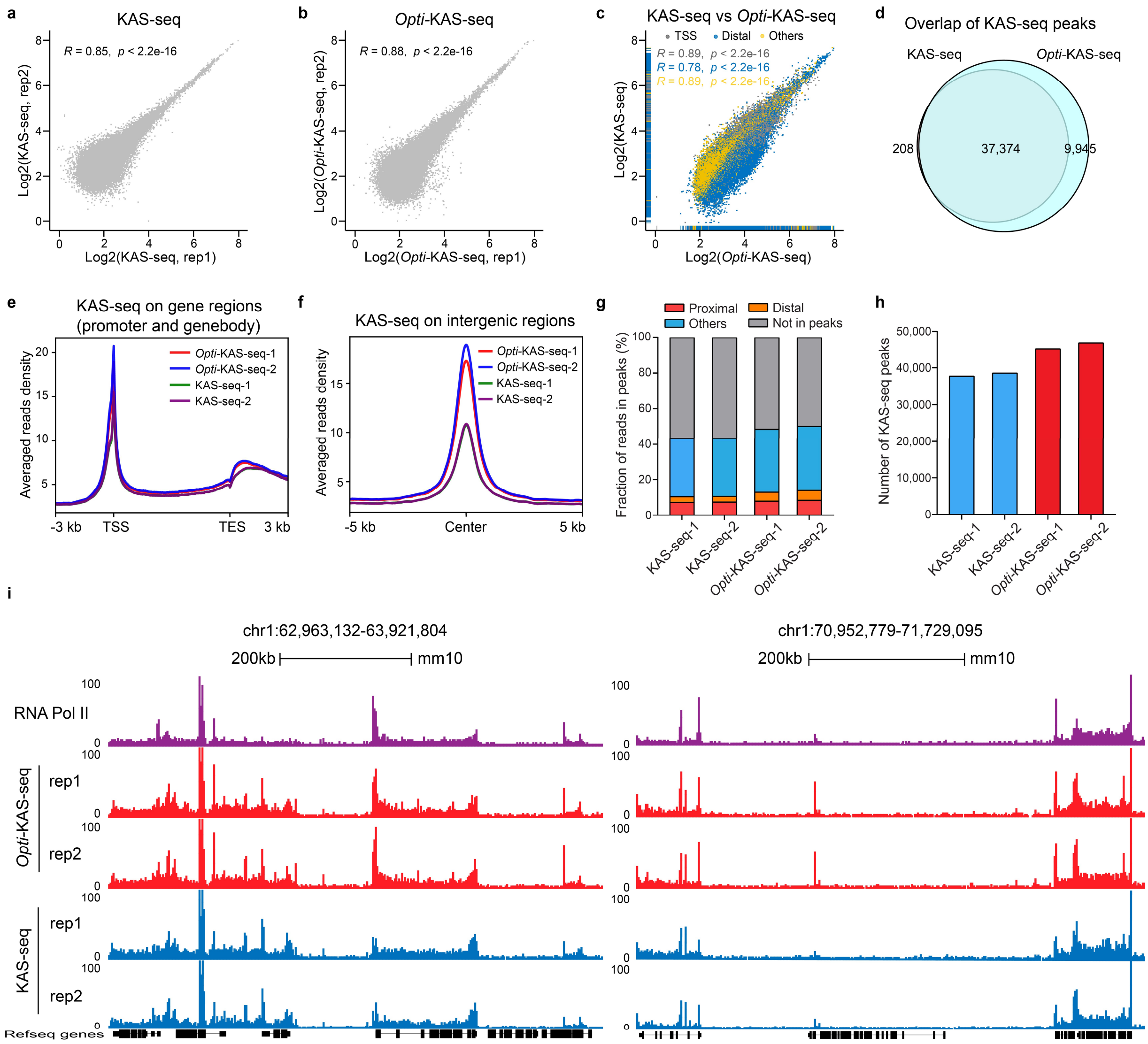

Supplementary Fig. 2: KAS-seq and *Opti*-KAS-seq datasets in mESCs.

a,**b**, Scatterplot showing the Pearson correlation between two replicates of KAS-seq (**a**) and *Opti*-KAS-seq (**b**) data in mESCs. Grey dots represent KAS-seq peaks (**a**, n = 64,019; **b**, n = 70,037). Both the Pearson correlation coefficients and significance (two-sided *p* values) are provided. **c**, Scatterplot comparing KAS-seq and *Opti*-KAS-seq data in mESCs across 1kb genomic bins on merged KAS-seq and *Opti*-KAS-seq peaks. Grey dots denote promoters (n = 13,890), blue dots denote distal CREs (n = 23,881), and yellow dots denote other genomic bins (n = 31,599) (as labeled). Both the Pearson correlation coefficients and significance (two-sided *p* values) are provided. **d**, Venn diagram illustrating the overlap of peaks identified using KAS-seq and *Opti*-KAS-seq datasets in mESCs. **e**,**f**, Metagene profiles showing the averaged read density of KAS-seq and *Opti*-KAS-seq datasets across gene-coding regions (**e**) (n = 22,868) and intergenic KAS-seq peaks (**f**) (n = 12,992) in mESCs. Gene-coding regions were defined as 3 kb upstream of TSS and 3 kb downstream of TES. **g**, Stacked bar plot showing the fraction of reads in peaks (FRiP)

that were mapped to promoters (±500 bp of TSS), distal cis-regulatory elements (distal CREs, >500 bp from TSS) and other regions from KAS-seq and *Opti*-KAS-seq datasets generated in mESCs. All values were determined from 30 million random aligned de-duplicated reads. **h**, Vertical bar plot showing the number of KAS-seq peaks from KAS-seq and *Opti*-KAS-seq datasets generated in mESCs. **i**, Snapshot of UCSC genome browser tracks displaying RNA Pol II ChIP-seq, KAS-seq, and *Opti*-KAS-seq data in mESCs across two representative regions. Two replicates for KAS-seq and *Opti*-KAS-seq datasets are displayed.

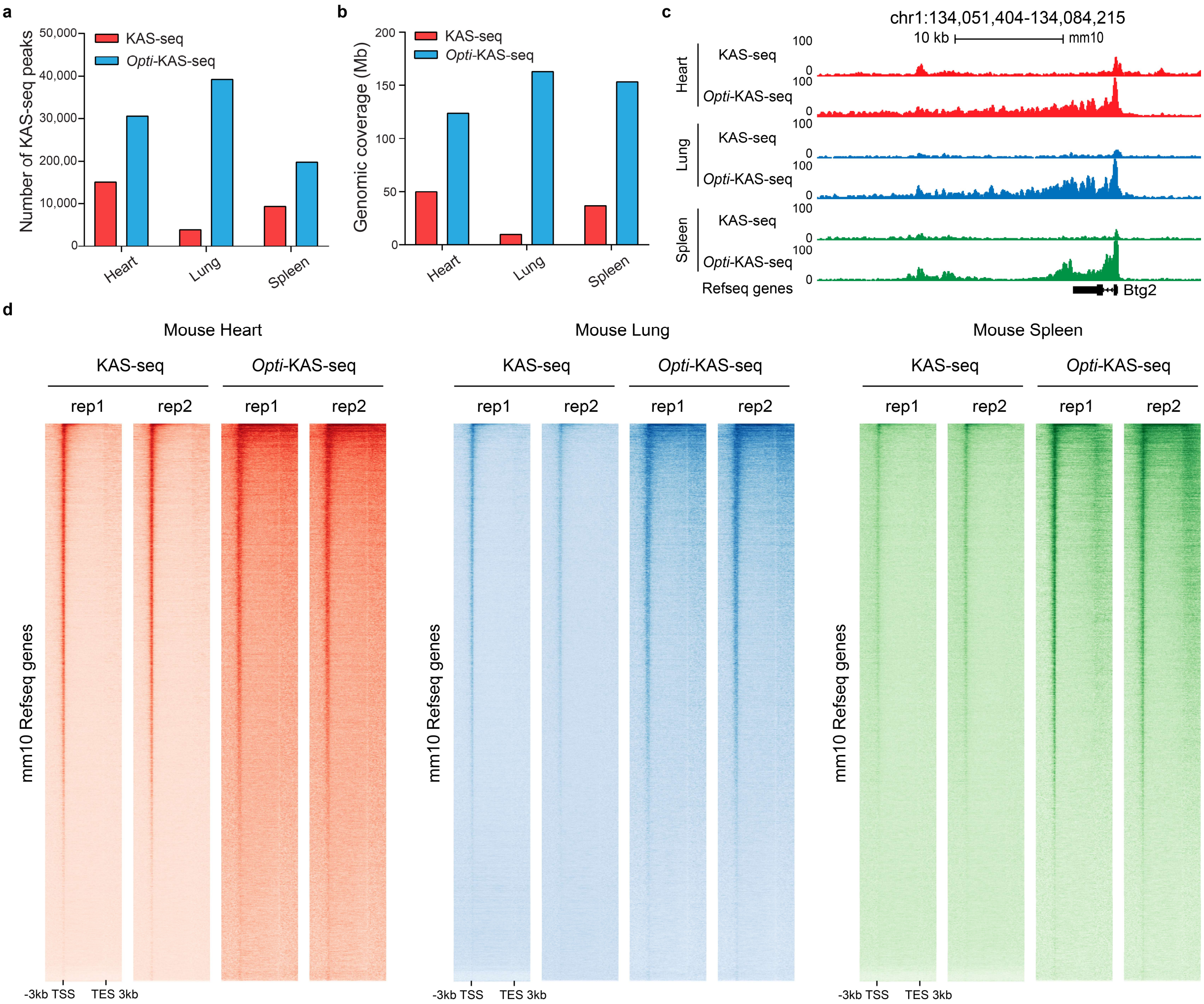

Supplementary Fig. 3: *Opti*-KAS-seq datasets were generated in three mouse tissue types that are not compatible with conventional KAS-seq protocol.

a, Grouped bar plot displaying the number of KAS-seq peaks from KAS-seq and *Opti*-KAS-seq datasets in mouse heart, lung, and spleen tissues. **b**, Grouped bar plot displaying the genomic coverage of KAS-seq peaks from KAS-seq and *Opti*-KAS-seq datasets generated in mouse heart, lung, and spleen tissues. **c**. Snapshot of UCSC genome browser tracks displaying KAS-seq and *Opti*-KAS-seq datasets generated in mouse heart, lung, and spleen tissues across a representative region (chr1:134,051,404-134,084,215). **d**, Heatmap plots showing the distribution of KAS-seq and *Opti*-KAS-seq read density at gene-coding regions (n = 22,868), with 3 kb upstream of TSS and 3 kb downstream of TES shown. KAS-seq and *Opti*-KAS-seq datasets were obtained from mouse heart (left panel), lung (middle panel), and spleen (right panel) tissues.

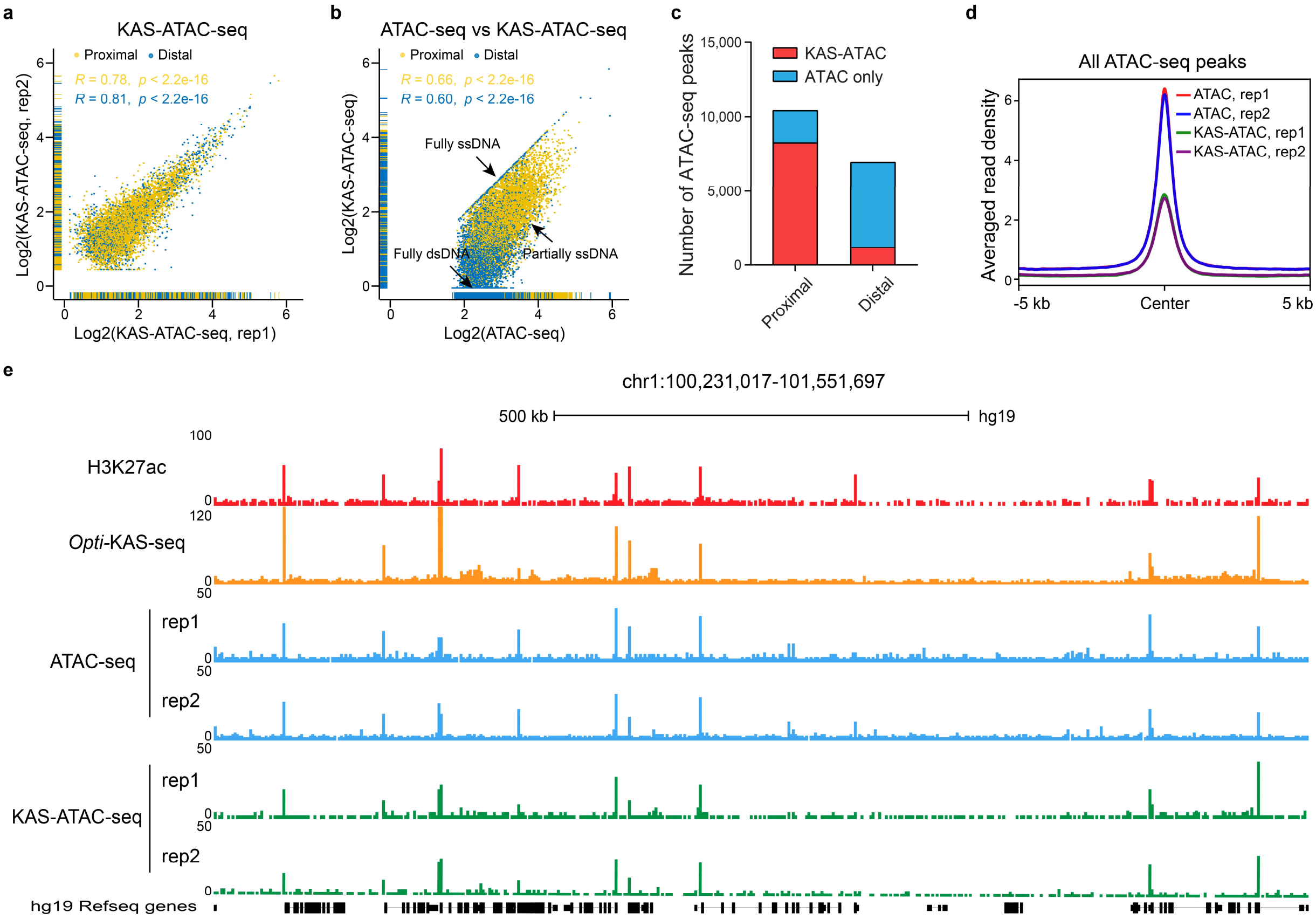

Supplementary Fig. 4: Implementation of KAS-ATAC-seq in HEK293T cells.

a, Scatterplot illustrating the Pearson correlation between two replicates of KAS-ATAC-seq data for proximal (n = 6,863) and distal (n = 1,445) peaks in fresh HEK293T cells. Yellow dots represent proximal peaks, blue dots represent distal peaks. Both the Pearson correlation coefficients and significance (two-sided *p* values) for proximal and distal peaks are provided. **b**, Scatterplot illustrating the Pearson correlation between ATAC-seq and KAS-ATAC-seq data for proximal (n = 9,410) and distal (n = 6,913) peaks in fresh HEK293T cells. Yellow dots represent proximal peaks, blue dots represent distal peaks. Both the Pearson correlation coefficients and significance (two-sided *p* values) for proximal and distal peaks are provided. Peaks exhibiting higher signals in KAS-ATAC-seq relative to ATAC-seq are normalized to the ATAC-seq signals. **c**, Stacked bar plot showing the numbers of ATAC-seq peaks, depicted by their overlap with KAS-ATAC-seq peaks, in HEK293T cells. Proximal and distal ATAC-seq peaks are shown separately. **d**, Metagene profile showing the distribution of averaged ATAC-seq and KAS-ATAC-seq read density across all ATAC-seq peaks identified in HEK293T cells, with 5 kb upstream and 5 kb downstream from the center of ATAC-seq peaks shown. **e**, Snapshot of UCSC genome browser tracks displaying H3K27ac ChIP-seq, *Opti*-KAS-seq, ATAC-seq, and KAS-ATAC-seq data in HEK293T cells on a representative region (chr1:100,231,017-101,551,697). Two replicates of KAS-seq and *Opti*-KAS-seq datasets are shown.

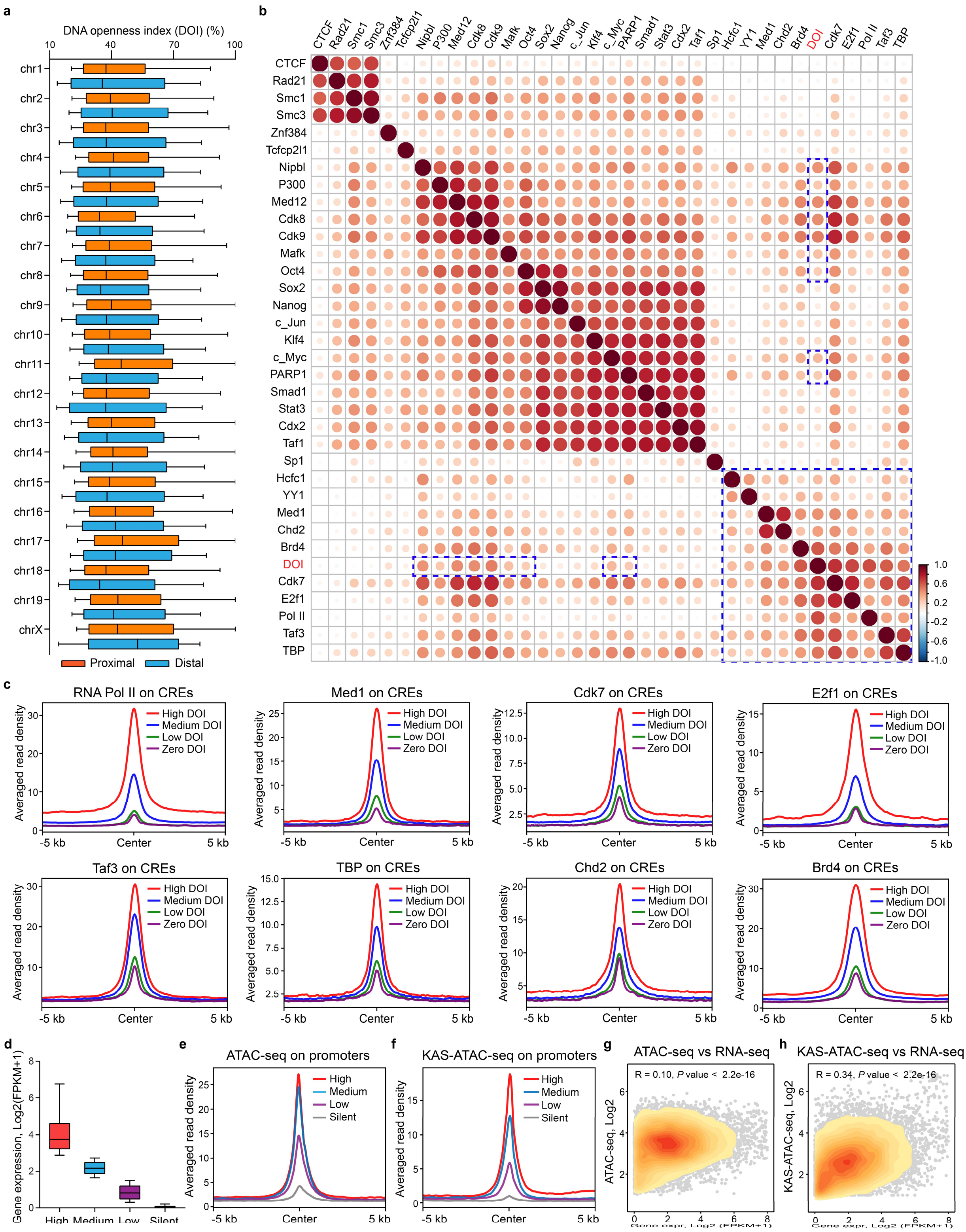

Supplementary Fig. 5: Specific transcription factors (TFs) are highly enriched at cis-regulatory elements (CREs) with high DNA openness index (DOI) in mESCs.

a, Horizontal boxplot illustrating the DNA openness index (DOI) of proximal and distal cis-regulatory elements (CREs) across different chromosomes. **b**, Heatmap illustrating the Pearson correlation between DNA openness index (DOI), RNA Pol II, and selected transcription factors (TFs) binding affinity at proximal cis-regulatory elements. TFs that have a Pearson correlation higher than 0.3 with the DNA openness index (DOI) are marked with blue dashed rectangle. The binding affinity of TFs on chromatin is quantified using ChIP-seq data. **c**, Metagene profiles showing the distribution of RNA Pol II and specific TFs (Med1, Cdk7, E2f1, Taf3, TBP, Chd2, and Brd4) binding density across four categories of proximal CREs in mESCs, with 5 kb upstream and 5 kb downstream from the center of CREs shown. These CREs are categorized based on different levels of DOI (High DOI, Medium DOI, Low DOI, and Zero DOI). **d**, Boxplot illustrating the classification of gene groups based on expression levels (FPKM, calculated using bulk RNA-seq data in mESCs). Genes with FPKM values above 0.5 are classified as expressed genes and then categorized into high, medium, and low expression groups, each group containing an equal number of genes (n = 3,822). Genes with FPKM values below 0.5 are classified as silent genes (n = 9,047). The box shows 1st quartile, median and 3rd quartile, respectively. **e**,**f**, Metagene profiles showing the distribution of averaged ATAC-seq (**e**) and KAS-ATAC-seq (**f**) read density at gene promoters with different expression levels (high, medium, low, silent) in mESCs, with 5 kb upstream and downstream of TSS shown. **g**,**h**, Scatterplots showing the Pearson correlation between gene expression (Gene expr, bulk RNA-seq) and chromatin accessibility from ATAC-seq (**g**), as well as ssDNA signals from KAS-ATAC-seq (**h**) on expressed genes (n = 11,467). The Pearson correlation coefficient (R) and associated two-sided *p* values are displayed at the top of the scatterplots. Points are color-coded in orange to indicate gene density.

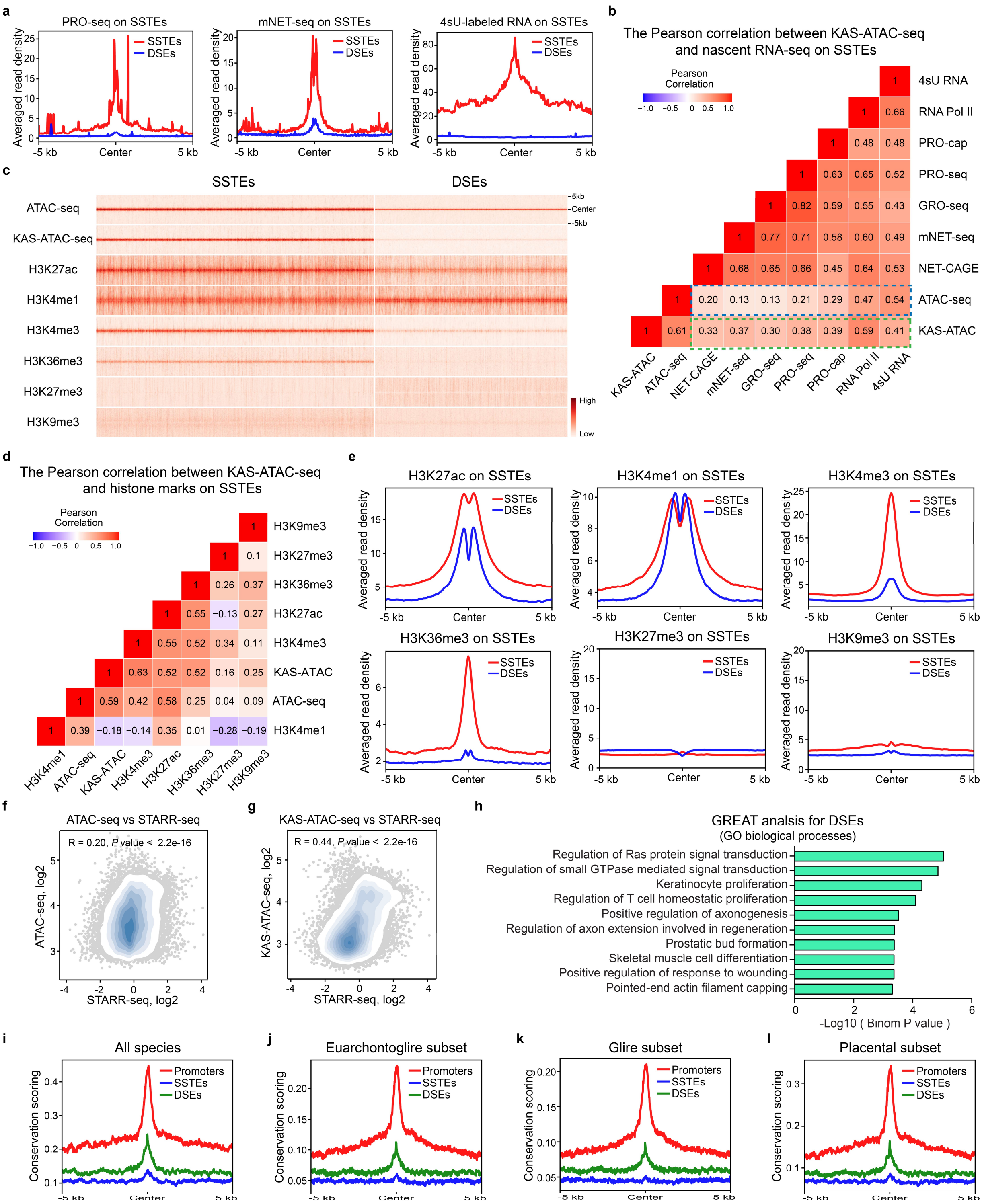

Supplementary Fig. 6: Correlation of KAS-ATAC-seq signals with nascent RNA transcription, histone marks, enhancer activities, and evolutionary conservation score on SSTEs in mESCs.

a, Metagene profiles showing nascent RNA transcriptional levels determined by various assays, including PRO-seq, mNET-seq, and 4sU RNA-seq, across SSTEs (red) and DSEs (blue), with 5 kb upstream and 5 kb downstream from the center of distal cis-regulatory elements (CREs) shown. **b**, Correlation heatmap illustrating the Pearson correlation coefficients to depict relationships among ATAC-seq, KAS-ATAC-seq, RNA Pol II binding, and nascent RNA transcription (4sU RNA, GRO-seq, PRO-seq, PRO-cap, mNET-seq, NET-CAGE) associated with SSTEs in mESCs. The Pearson correlation coefficients were labeled on the correlation heatmap. Pearson correlation coefficients between ATAC-seq signals and nascent RNAs levels are marked with blue dashed rectangles, and while Pearson correlation coefficients between KAS-ATAC-seq signals and RNA transcription levels are marked with green dashed rectangles. **c**, Horizontal heatmap showing the enrichment of ATAC-seq, KAS-ATAC-seq, and various histone marks ChIP-seq read density on SSTEs (top panel) and DSEs (bottom panel) in mESCs. Regions spanning 5 kb upstream and 5 kb downstream from the center of distal CREs are shown. **d**, Correlation heatmap illustrating the Pearson correlation coefficients among ATAC-seq, KAS-ATAC-seq, and various histone marks (H3K27ac, H3K4me1, H3K4me3, H3K36me3, H3K27me3, and H3K9me3) ChIP-seq data on SSTEs in mESCs. The Pearson correlation coefficients were labeled on the correlation heatmap. **e**, Metagene profiles showing the averaged read density of various histone marks ChIP-seq data (H3K27ac, H3K4me1, H3K4me3, H3K36me3, H3K27me3, and H3K9me3) over SSTEs (red) and DSEs (blue) in mESCs. **f**,**g**, Scatterplots showing the Pearson correlation between STARR-seq and ATAC-seq (**f**) or KAS-ATAC-seq (**g**) datasets on SSTEs (n = 18,783). The Pearson correlation coefficients (R) and associated two-sided *p* values are displayed at the top of the scatterplots. Points are color-coded in light blue to indicate SSTE density. **h**, Horizontal bar plot illustrating the Gene Ontology (GO) biological processes derived from the GREAT analysis of DSEs in mESCs. The significance of each process is calculated using the binomial test. **i**,**j**,**k**,**l**, Metagene profiles showing the averaged read density of evolutionary conservation scores obtained through phyloP methods, for all species and three subsets across promoters (red), SSTEs (blue) and DSEs (green), with 5 kb upstream and 5 kb downstream from the center of CREs shown.

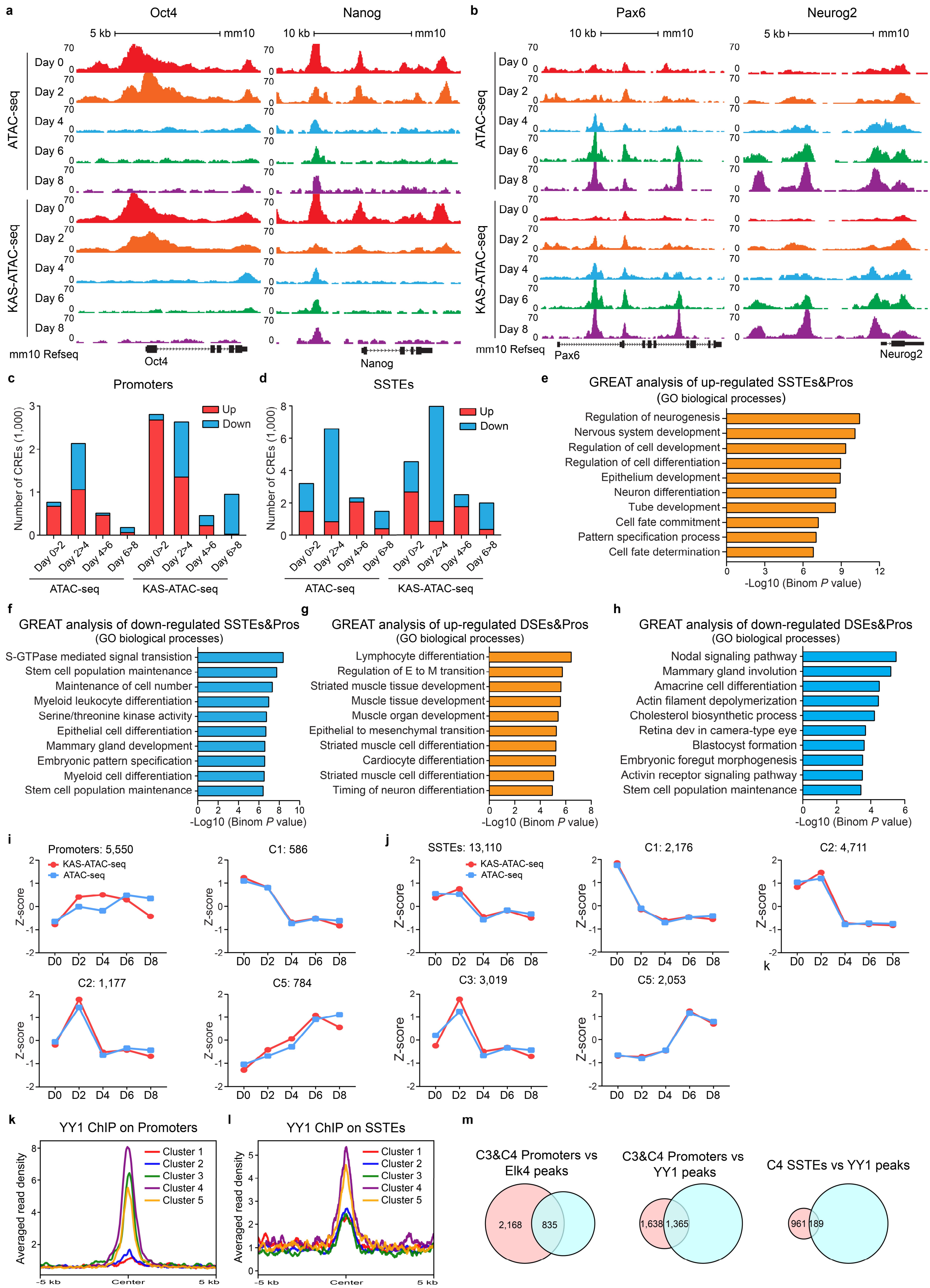

a
Oct4
Nanog
b
Pax6
Neurog2
ATAC-seq
KAS-ATAC-seq
Day 0
Day 2
Day 4
Day 6
Day 8
mm10 Refseq
c
Promoters
Up
Down
Number of CREs (1,000)
ATAC-seq
KAS-ATAC-seq
d
SSTEs
e
GREAT analysis of up-regulated SSTEs&Pros
(GO biological processes)
Regulation of neurogenesis
Nervous system development
Regulation of cell development
Regulation of cell differentiation
Epithelium development
Neuron differentiation
Tube development
Cell fate commitment
Pattern specification process
Cell fate determination
-Log10 (Binom P value)
f
GREAT analysis of down-regulated SSTEs&Pros
(GO biological processes)
S-GTPase mediated signal transition
Stem cell population maintenance
Maintenance of cell number
Myeloid leukocyte differentiation
Serine/threonine kinase activity
Epithelial cell differentiation
Mammary gland development
Embryonic pattern specification
Myeloid cell differentiation
Stem cell population maintenance
g
GREAT analysis of up-regulated DSEs&Pros
(GO biological processes)
Lymphocyte differentiation
Regulation of E to M transition
Striated muscle tissue development
Muscle tissue development
Muscle organ development
Epithelial to mesenchymal transition
Striated muscle cell differentiation
Cardiocyte differentiation
Striated muscle cell differentiation
Timing of neuron differentiation
h
GREAT analysis of down-regulated DSEs&Pros
(GO biological processes)
Nodal signaling pathway
Mammary gland involution
Amacrine cell differentiation
Actin filament depolymerization
Cholesterol biosynthetic process
Retina dev in camera-type eye
Blastocyst formation
Embryonic foregut morphogenesis
Activin receptor signaling pathway
Stem cell population maintenance
i
Promoters: 5,550
KAS-ATAC-seq
ATAC-seq
C1: 586
C2: 1,177
C5: 784
Z-score
j
SSTEs: 13,110
C1: 2,176
C2: 4,711
C3: 3,019
C5: 2,053
k
YY1 ChIP on Promoters
Averaged read density
Cluster 1
Cluster 2
Cluster 3
Cluster 4
Cluster 5
-5 kb
Center
5 kb
l
YY1 ChIP on SSTEs
m
C3&C4 Promoters vs Elk4 peaks
2,168
835
C3&C4 Promoters vs YY1 peaks
1,638
1,365
C4 SSTEs vs YY1 peaks
961
189

Supplementary Fig. 7: KAS-ATAC-seq accurately captures the transcriptional dynamics of promoters and SSTEs during the neural differentiation of mESCs into NPCs.

a,**b**, Snapshots of UCSC genome browser tracks showing ATAC-seq and KAS-ATAC-seq data for stem cell pluripotency genes (*Oct4* and *Nanog*) (**a**) and early neural marker genes (*Pax6* and *Neurog2*) (**b**), at specific time point during mouse neural differentiation from mESCs to NPCs. **c**,**d**, Stacked bar plots showing the number of dynamic promoters (**c**) and SSTEs (**d**) identified using ATAC-seq and KAS-ATAC-seq data at different time-points compared to the previous time-point during in vitro neural differentiation from mESCs to NPCs. **e**,**f**, Horizontal bar plot illustrating the Gene Ontology (GO) biological processes derived from the GREAT analysis of up- (**e**) and down-regulated (**f**) ssDNA promoters and SSTEs identified using KAS-ATAC-seq data during mouse neural differentiation from mESCs to NPCs. The significance of each process is calculated using the binomial test. **g**,**h**, Horizontal bar plot illustrating the Gene Ontology (GO) biological processes derived from the GREAT analysis of up- (**g**) and down-regulated (**h**) non-ssDNA promoters and DSEs identified using ATAC-seq and KAS-ATAC-seq data during mouse neural differentiation from mESCs to NPCs. The significance of each process is calculated using the binomial test. **i**, Line graph depicting the z-scores of all, C1, C2, and C5 promoters in the clustered heatmap of dynamic promoters. The z-scores were calculated using ATAC-seq data (blue) and KAS-ATAC-seq data (red) at various stages of mouse neural differentiation. **j**, Line graph depicting the z-scores of all, C1, C2, C3, and C5 SSTEs in the clustered heatmap of dynamic SSTEs. The z-scores were calculated using ATAC-seq data (blue) and KAS-ATAC-seq data (red) at various stages of mouse neural differentiation. **k**,**l**, Metagene profiles showing the averaged read density of YY1 ChIP-seq data over different ssDNA promoters (**k**) and SSTEs (**l**) clusters in the clustered heatmaps during mouse neural differentiation. **m**, Venn plots showing the overlap analysis between C3&C4 promoters and Elk4 peaks (left panel), C3&C4 promoters and YY1 peaks (middle panel), and C4 SSTEs and YY1 peaks (right panel).

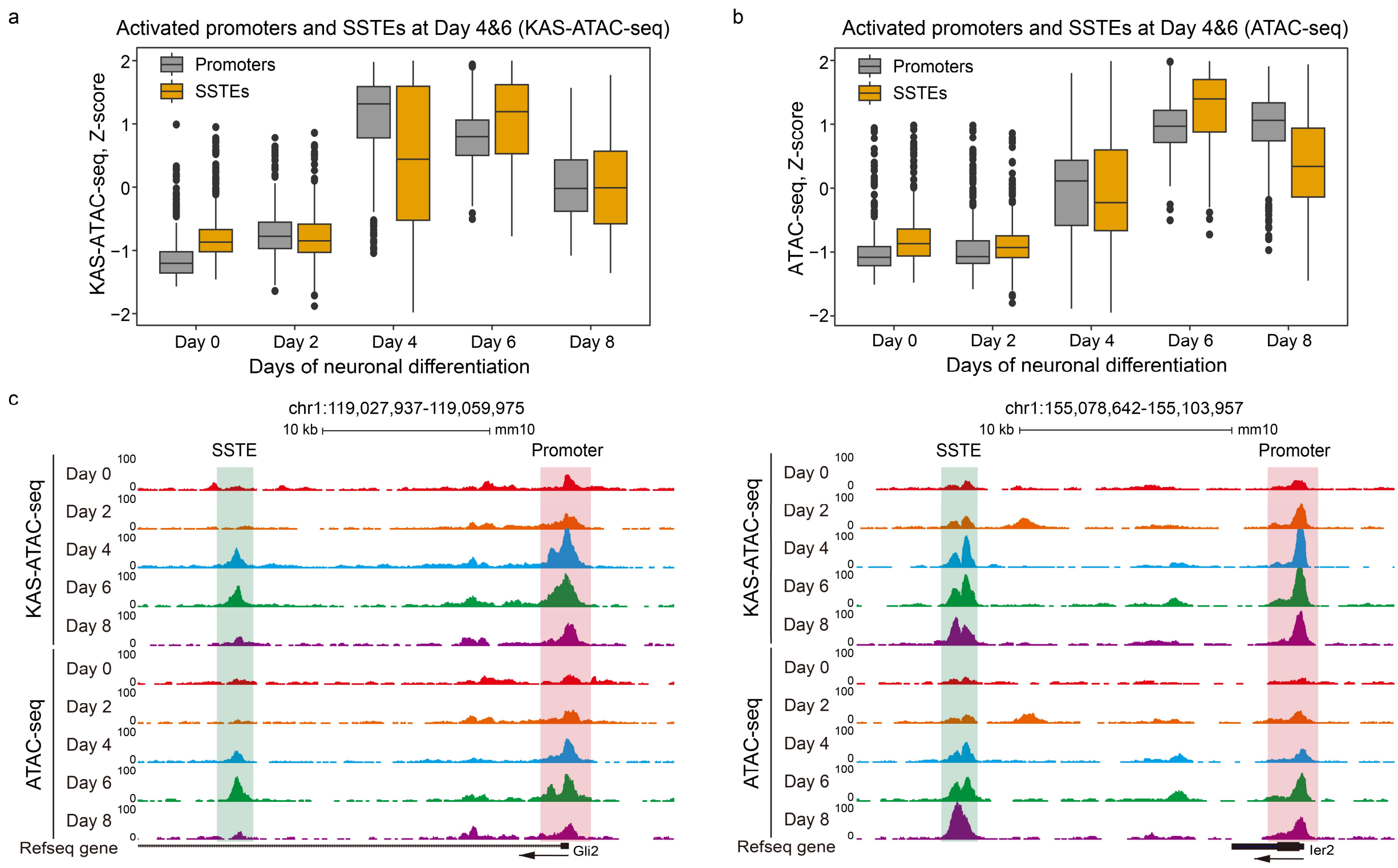

Supplementary Fig. 8: The activation timing of promoters and SSTEs during mouse neural differentiation induced by retinoic acid (RA) treatment.

a, Grouped boxplot showing z-scores of activated promoters (grey boxes) and SSTEs (yellow boxes) identified on Days 4 and 6 of neural differentiation after RA treatment. Z-scores were calculated using KAS-ATAC-seq data from Day 0 and 8 during mouse neural differentiation of mESCs to NPCs. **b**, Grouped boxplot showing z-scores of activated promoters (grey boxes) and SSTEs (yellow boxes) identified on Days 4 and 6 of neural differentiation after RA treatment. Z-scores were calculated using ATAC-seq data from Days 0 and 8 during mouse neural differentiation of mESCs to NPCs. **c**, Snapshots of UCSC genome browser tracks showing ATAC-seq and KAS-ATAC-seq data on the promoters and SSTEs of two example genes, *Gli2* (left panel) and *Ler2* (right panel), at specific time point from Day 0 to Day 8 during mouse neural differentiation from mESCs to NPCs.

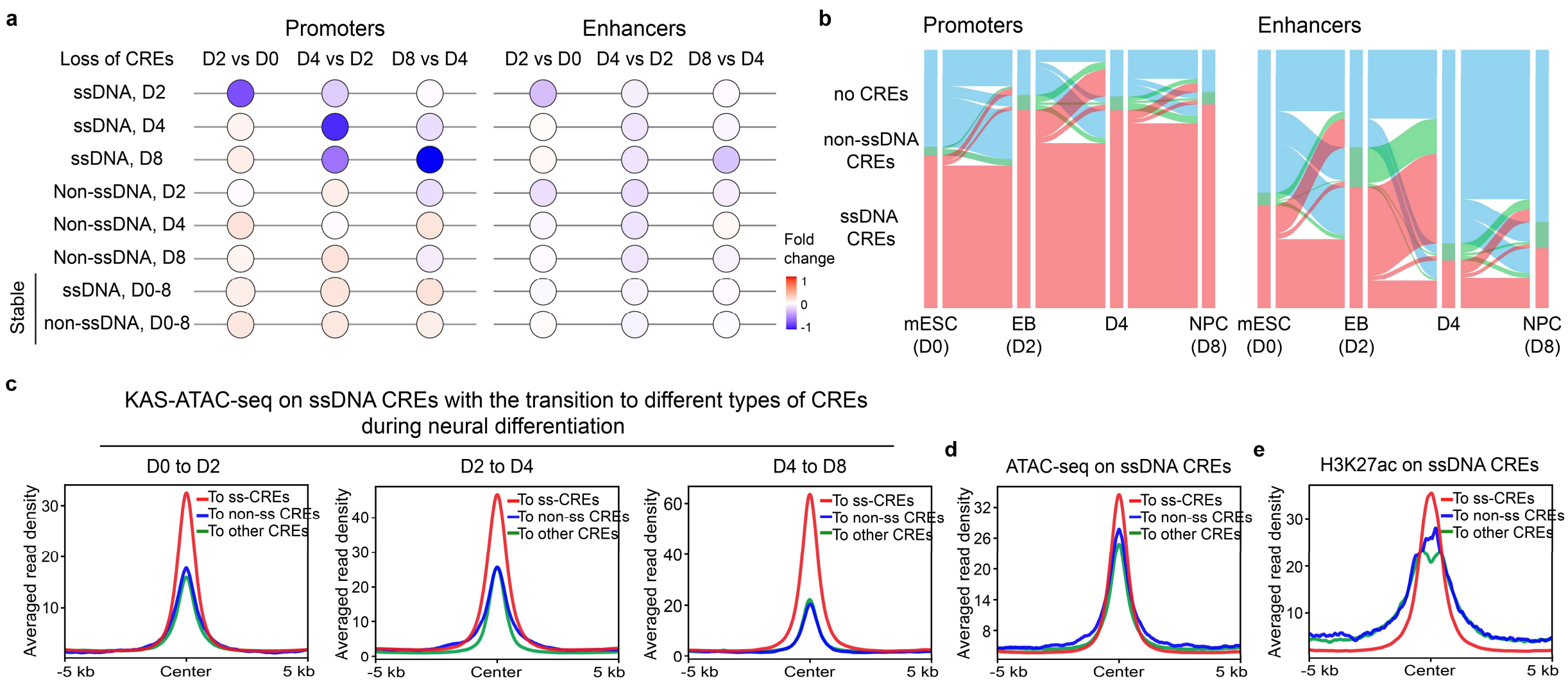

Supplementary Fig. 9: The transition of ssDNA promoters and SSTEs during the neural differentiation of mESCs into NPCs.

a, Bubble plots showing the fold changes of gene expression levels between consecutive stages for genes associated with “loss of” and stable (no-change) ssDNA and non-ssDNA CREs defined by KAS-ATAC-seq and ATAC-seq data during the neural differentiation from mESCs to EBs and NPCs (D0 to D8). CREs are shown separately as promoters and enhancers. The color key, ranging from blue to red, indicates the median of fold changes of gene expression levels from low to high, respectively. The bulk cell RNA-seq data are referred from GSE151900. **b**, Alluvial plots showing the transition of dynamic promoters (left) and enhancers (right) during the neural differentiation. Each line in the plot represents a dynamic promoter or enhancer, and the total dynamic CREs shown are those classified as dynamic promoter or enhancer in at least one of the analyzed stages. **c**, Metagene profiles showing the averaged read density of KAS-ATAC-seq data over three groups of ssDNA CREs transitioning into various CRE subtypes (to ssDNA CREs, to non-ssDNA CREs, and to other CREs) in later stage of neural differentiation from mESCs to NPCs. **d**,**e**, Metagene profiles showing the averaged read density of ATAC-seq (**d**) and H3K27ac (**e**) ChIP-seq data in mESCs over three groups of SSTEs transitioning into various CRE subtypes (to ssDNA CREs, to non-ssDNA CREs, and to other CREs) during the neural differentiation from mESCs to NPCs.

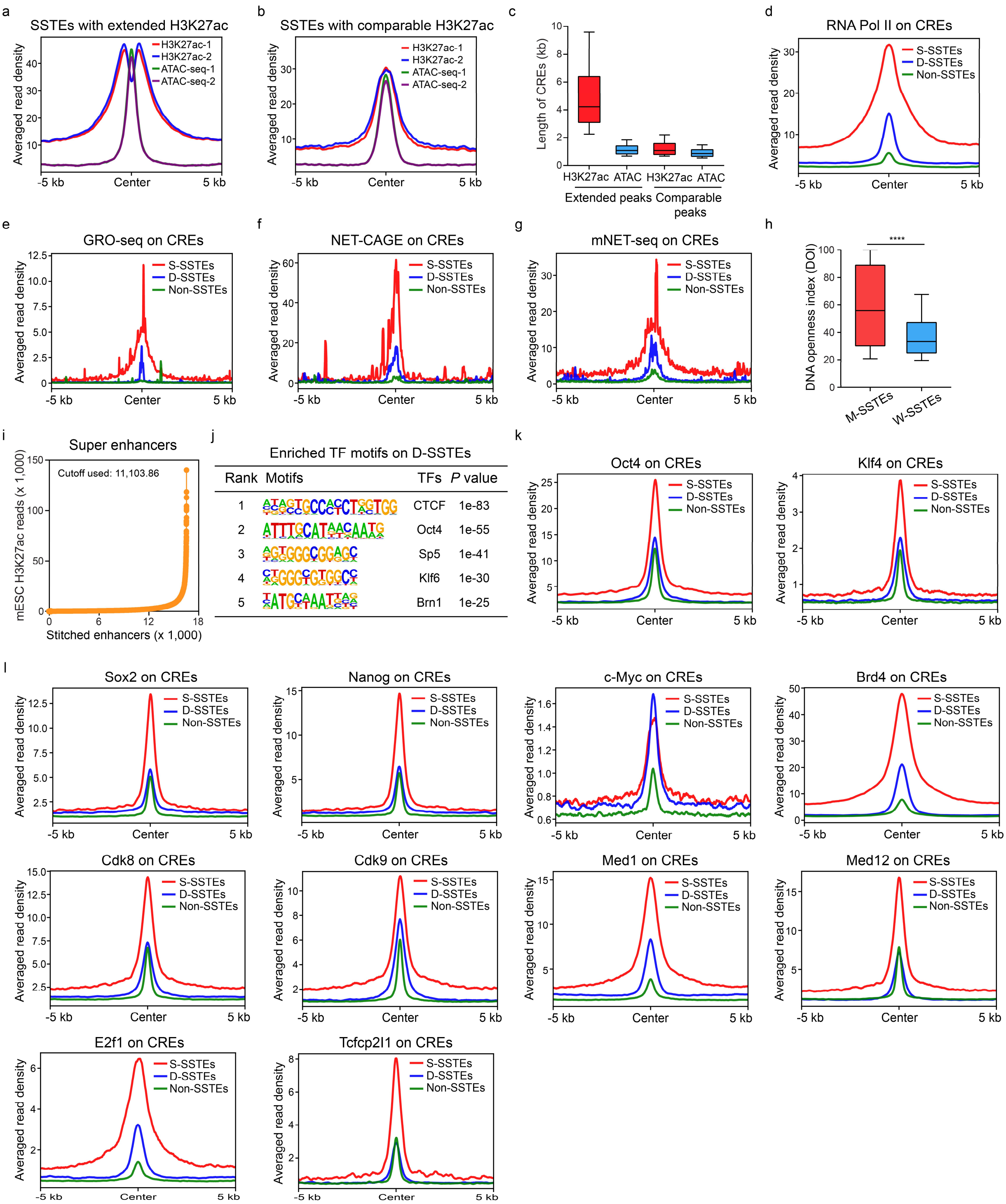

Supplementary Fig. 10: Distinct characteristics and functional implications of two types of SSTE (S- and D-SSTEs) in mESCs.

a,**b**, Metagene profiles showing the averaged read density of H3K27ac ChIP-seq and KAS-ATAC-seq data across SSTEs with extended H3K27ac peaks (**a**), as well as comparable H3K27ac peaks

(**b**) in mESCs. Regions spanning 5 kb upstream and 5 kb downstream from the center of cis-regulatory elements (CREs) are shown. **c**, Box plot comparing the length of H3K27ac, and KAS-ATAC-seq peaks on SSTEs with extended H3K27ac peaks, as well as comparable H3K27ac peaks in mESCs. The box shows 1st quartile, median and 3rd quartile, respectively. **d**,**e**,**f**,**g**, Metagene profiles showing the averaged read density of RNA Pol II (**d**), GRO-seq (**e**), NET-CAGE (**f**), and mNET-seq (**g**) data across S-SSTEs, D-SSTEs, and DSEs in mESCs. Regions spanning 5 kb upstream and 5 kb downstream from the center of SSTEs are shown. **h**, Boxplot comparing the DNA openness index (DOI) of S-SSTEs and D-SSTEs in mESCs. A paired student t-test was used to calculate the *p* value. *P* value < 0.0001 was shown as ****. The box shows 1st quartile, median and 3rd quartile, respectively. Whiskers extend to show the range from the 10th to the 90th percentile, with data points outside this range not shown. **i**, Distribution visualization of H3K27ac ChIP-Seq signals identifying 2,646 super enhancers (SEs) in mESCs. Enhancers are arranged in ascending order based on their input-normalized H3K27ac ChIP-Seq intensities. Super-enhancers are defined as the population of enhancers above the inflection point (H3K27ac ChIP-Seq intensities cutoff) of the curve. **j**, Tables presenting the enriched transcription factors (TFs) motifs identified on D-SSTEs in mESCs. The hypergeometric test was used to calculate the p values. **k**,**l**, Metagene profiles showing the averaged read density of specific transcription factors (Oct4, Sox2, Nonag, c-Myc, Klf4, Brd4, Med1, Med12, Cdk8, Cdk9, E2f1, and Tcfcp2l1) ChIP-seq data across S-SSTEs, D-SSTEs, and DSEs in mESCs. Regions spanning 5 kb upstream and 5 kb downstream from the center of regulatory elements are shown.

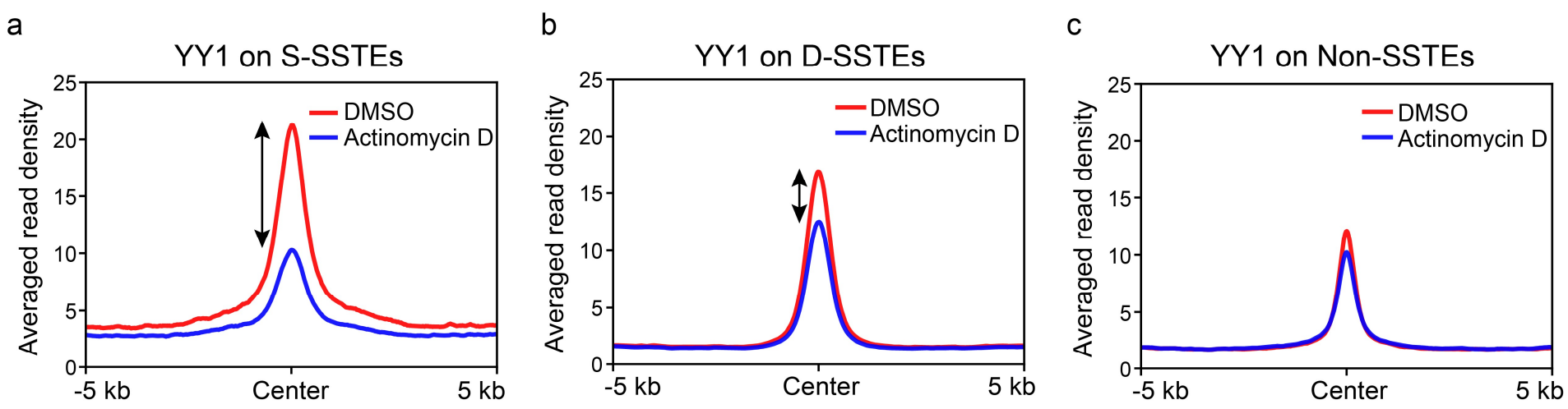

Supplementary Fig. 11: Transcription perturbation by Actinomycin D treatment has varying effects on YY1 binding to different types of SSTEs in mESCs.

a,**b**,**c**, Metagene profiles showing the averaged read density of YY1 ChIP-seq data obtained from DMSO (red) or Actinomycin D (blue) (transcription inhibition) treated mESCs, across S-SSTEs (**a**), D-SSTEs (**b**), and DSEs (**c**). Regions spanning 5 kb upstream and 5 kb downstream from the center of cis-regulatory elements (CREs) are shown.

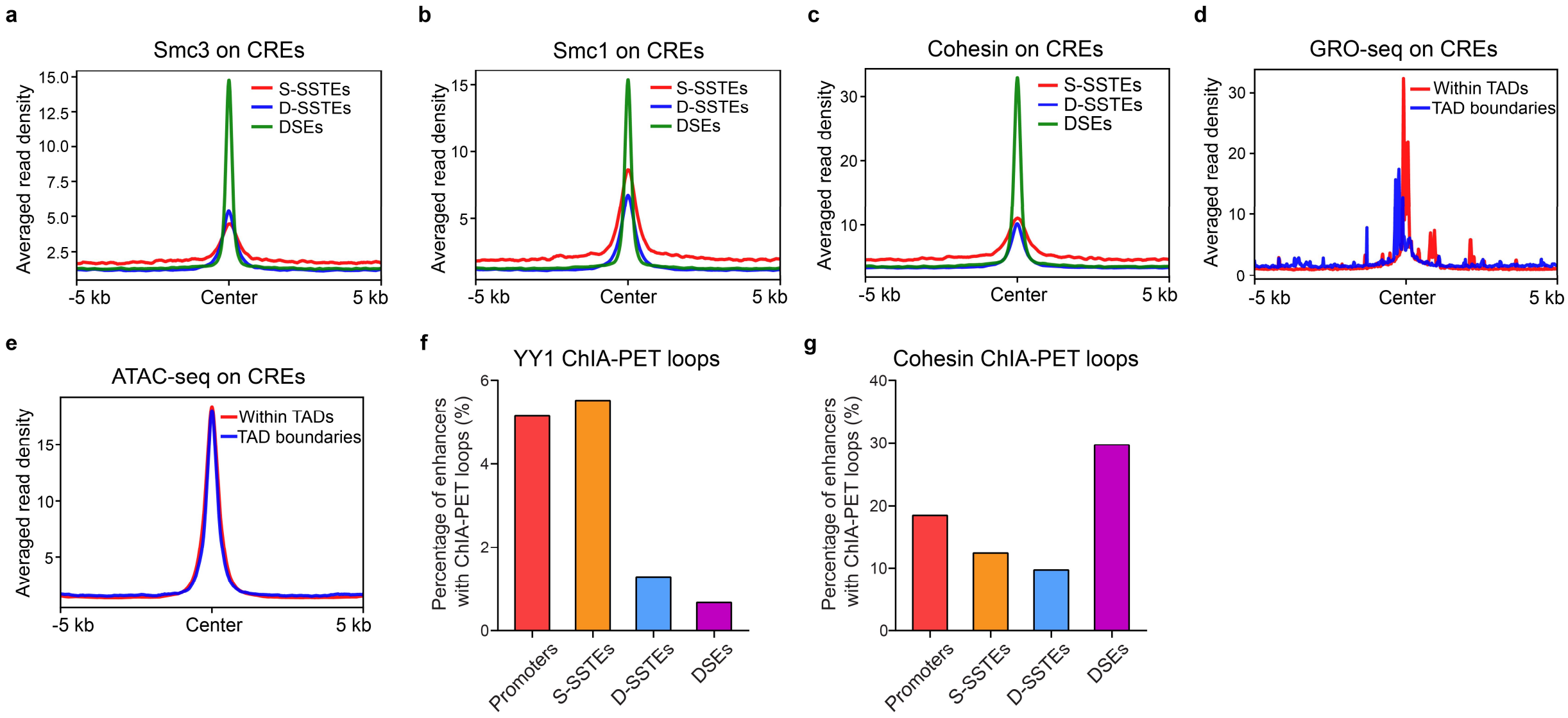

Supplementary Fig. 12: Interaction between the Cohesin complex and associated ChIA-PET loops across different SSTE subtypes in mESCs.

a,**b**,**c**, Metagene profiles showing the averaged read density of Smc3 (**a**), Smc1 (**b**), and Cohesin (**c**) ChIP-seq data across S-SSTEs, D-SSTEs, and DSEs in mESCs. **d**,**e**, Metagene profiles showing the averaged read density of GRO-seq (**d**) and ATAC-seq (**e**) data across SSTEs within TADs, or at TAD boundaries in mESCs. **f**,**g**, Vertical bar plot illustrating the percentages of promoters, S-SSTEs, D-SSTEs, and DSEs associated with long-range interaction loops defined by YY1 (**f**) and Cohesin (**g**) ChIA-PET data in mESCs.
