# Supplementary material for: Quantitative analysis of cis-regulatory elements in transcription with KAS-ATAC-seq
Source: Nat Commun. 2024 Aug 10;15:6852. doi: 10.1038/s41467-024-50680-8 (PMC11316786; doi:10.1038/s41467-024-50680-8)
Supplement: Supplementary file 2 [file 41467_2024_50680_MOESM2_ESM.pdf]

nature portfolio

Peer Review File

Quantitative analysis of cis-regulatory elements in transcription with KAS-ATAC-seq

Open Access This file is licensed under a Creative Commons Attribution 4.0 International License, which permits use, sharing, adaptation, distribution and reproduction in any medium or format, as long as you give appropriate credit to the original author(s) and the source, provide a link to the Creative Commons license, and indicate if changes were made. In the cases where the authors are anonymous, such as is the case for the reports of anonymous peer reviewers, author attribution should be to 'Anonymous Referee' followed by a clear attribution to the source work. The images or other third party material in this file are included in the article's Creative Commons license, unless indicated otherwise in a credit line to the material. If material is not included in the article's Creative Commons license and your intended use is not permitted by statutory regulation or exceeds the permitted use, you will need to obtain permission directly from the copyright holder. To view a copy of this license, visit http://creativecommons.org/licenses/by/4.0/.

REVIEWER COMMENTS

Reviewer #1 (Remarks to the Author):

In their manuscript, "Quantitative analysis of cis-regulatory elements in transcription with KAS-ATAC-seq," Lyu et al develop a version of their kethoxal-assisted sequencing (KAS-seq) approach that combines elements of hyperactive Tn5-based accessibility profiling with single-stranded DNA sequencing, to provide more mechanistic insight into bulk-averaged chromatin accessibility signal as measured by the now-standard ATAC-seq assay. I have very few comments – I think this is an interesting methods paper well suited for Nature Communications. I just have a few suggestions / questions for the authors that might involve some re-analyses and text changes.

Comments

• Can the authors expand on why the Accel-NGS Methyl-seq kit is optimal for the Opti-KAS-seq library preparation? There are several approaches for sequencing single-stranded DNA (see PMID28024297 and PMID30811994, for example) – what are some of the design decisions for using this kit for Opti-KAS-seq?

• Can the authors integrate KAS-ATAC-seq measurements with Pol II pausing index (as measured by either mNET-seq or PRO-seq). Does the extent of ssDNA accumulation track with Pol II pausing?

Reviewer #2 (Remarks to the Author):

In this manuscript, Lyu, Gao, and Wu et al. described developing an innovative approach for the analysis of cis-regulatory elements (CREs). The authors first developed an optimized KAS-seq (Opti-KAS-seq) with significantly improved performances in analyzing primary cells and tissue samples. Next, they integrated the Opti-KAS-seq with ATAC-seq and further developed the KAS-ATAC-seq that identifies the location of CREs and measures their activity states simultaneously. To demonstrate the utilities of the new method, they further applied it to mESCs differentiation into NPCs and uncovered different groups of CREs with distinct regulatory roles during this process. The investigation is comprehensive, the data presentation is clear, and the manuscript is well-written. This method will be a timely contribution to the epigenomics community.

A few minor suggestions for the current manuscript:

1. What is the required sequencing depth for Opti-KAS-seq? Could the authors perform a power analysis (i.e., downsample the #s of sequenced reads) for peak calling comparisons between Opti-KAS-seq and KAS-seq?

2. In Line 190 and Supplementary Fig. 5b, it is a bit unclear what the "correlations" between identified proteins and DOI are. Are they TF binding motifs enrichment scores, genome-wide read distributions/colocalization of protein ChIP-seq reads and DOI, or other measurements?

3. Lines 273-276, the earlier activated promoter and SSTEs are activated on Days 4 & 6 (for promoters) and Day 4 (for SSTEs). Does this suggest distal enhancers are activated prior to promoters? Or is there also a portion of CREs (but in a lower percentage compared to the promoters C3&4 and SSTEs C4) that are activated earlier in KAS-ATAC-seq?

4. The different roles of SSTEs and DSEs in TAD boundaries are exciting. Could the author discuss the feasibility of using KAS-ATAC-seq & ATAC-seq data to identify CREs & TAD boundaries?

Point-by-point response

Summary of Revision

We are grateful to all reviewers for their thoughtful and constructive comments to our manuscript, which have significantly improved its quality. All changes in the manuscript are marked in blue. The following is a summary of key experiments and analyses we have performed during revision:

1. We constructed *Opti*-KAS-seq libraries in HEK293T cells using both the Swift Accel-NGS Methyl-seq kit and the KAPA HyperPrep kit. Our comparative analysis revealed that the Swift Accel-NGS Methyl-seq kit outperforms the KAPA HyperPrep kit in terms of overall efficiency for detecting ssDNA using *Opti*-KAS-seq.

2. We conducted a correlation analysis using *Opti*-KAS-seq, KAS-ATAC-seq, RNA Pol II ChIP-seq, and the pausing index calculated from *Opti*-KAS-seq, PRO-seq, and mNET-seq. Our results showed that KAS-ATAC-seq has a low correlation with the pausing index from PRO-seq and mNET-seq. Additionally, both *Opti*-KAS-seq and KAS-ATAC-seq correlate well with RNA Pol II ChIP-seq data, indicating that ssDNA accumulation on promoters detected by these methods is linked to RNA Pol II pausing.

3. We conducted a power analysis by down-sampling the number of uniquely mapped reads from KAS-seq and *Opti*-KAS-seq data generated in HEK293T cells. Our results indicate that *Opti*-KAS-seq requires significantly less sequencing depth than KAS-seq to achieve robust peak calling. For optimal ssDNA enrichment in practical applications of *Opti*-KAS-seq, we recommend a minimum sequencing depth of 40 million uniquely mapped reads.

4. To investigate the activation timing of promoters and SSTEs during neural differentiation induced by RA treatment, we defined activated promoters and enhancers on Days 4 and 6 using KAS-ATAC-seq and ATAC-seq data, respectively, and tracked their dynamics from Day 0 to Day 8. Our results show that promoters and SSTEs were activated simultaneously during RA treatment. Additionally, we found that some promoters and SSTEs were activated earlier in KAS-ATAC-seq than in ATAC-seq.

Reviewer #1:

Comments for the Author:

In their manuscript, "Quantitative analysis of cis-regulatory elements in transcription with KAS-ATAC-seq," Lyu et al develop a version of their kethoxal-assisted sequencing (KAS-seq) approach that combines elements of hyperactive Tn5-based accessibility profiling with single-stranded DNA sequencing, to provide more mechanistic insight into bulk-averaged chromatin accessibility signal as measured by the now-standard ATAC-seq assay. I have very few comments – I think this is an interesting methods paper well suited for Nature Communications. I just have a few suggestions / questions for the authors that might involve some re-analyses and text changes.

Response: We are grateful for the reviewer's appreciation of this work, suggestions, and constructive comments.

Major points:

1. Can the authors expand on why the Accel-NGS Methyl-seq kit is optimal for the *Opti*-KAS-seq library preparation? There are several approaches for sequencing single-stranded DNA (see PMID28024297 and PMID30811994, for example) – what are some of the design decisions for using this kit for *Opti*-KAS-seq?

Response: We appreciate the reviewer's comment regarding the choice of the Swift Accel-NGS Methyl-seq kit for the *Opti*-KAS-seq library preparation. The rationale behind selecting this kit is grounded in a comparative analysis conducted for KAS-seq data generated in HEK293T cells using both the Swift Accel-NGS Methyl-seq kit (Swift kit) and the KAPA HyperPrep kit (KAPA kit). The Swift kit employs a highly efficient single-stranded DNA (ssDNA) adapter ligation-based method, making it ideal for preparing NGS libraries of ssDNA fragments. This kit has demonstrated excellent performance with BS-treated samples[1]. In contrast, the KAPA HyperPrep kit uses a double-stranded DNA (dsDNA) adapter ligation-based method, making it suitable for high-throughput NGS library construction[2].

Our comparative analysis revealed that the Swift library identified significantly more KAS-seq peaks (70,515) compared to the KAPA library (57,457), with a higher number of unique peaks (22,579 vs. 10,888) and substantial overlap (47,936) (Response Fig. 1a), indicating superior sensitivity in detecting KAS-seq peaks. The Swift library consistently showed higher ssDNA read density at transcription start sites (TSS), gene bodies, transcription end sites (TES) (Response Fig. 1b), and centers of intergenic KAS-seq peaks (Response Fig. 1c), suggesting enhanced enrichment and capture efficiency of ssDNA regions. Additionally, the Swift library had a higher proportion of reads mapping to promoters, distal elements, and other genomic features (Response Fig. 1d). UCSC genome browser views of representative regions further supported the superior performance of the Swift Library, with more pronounced and distinct peaks (Response Fig. 1e).

Collectively, these observations underscore that the ssDNA ligation-based method of the Swift Accel-NGS Methyl-seq kit provides superior performance in terms of

sensitivity, specificity, and overall efficiency for KAS-seq library construction, justifying its selection over the KAPA HyperPrep kit.

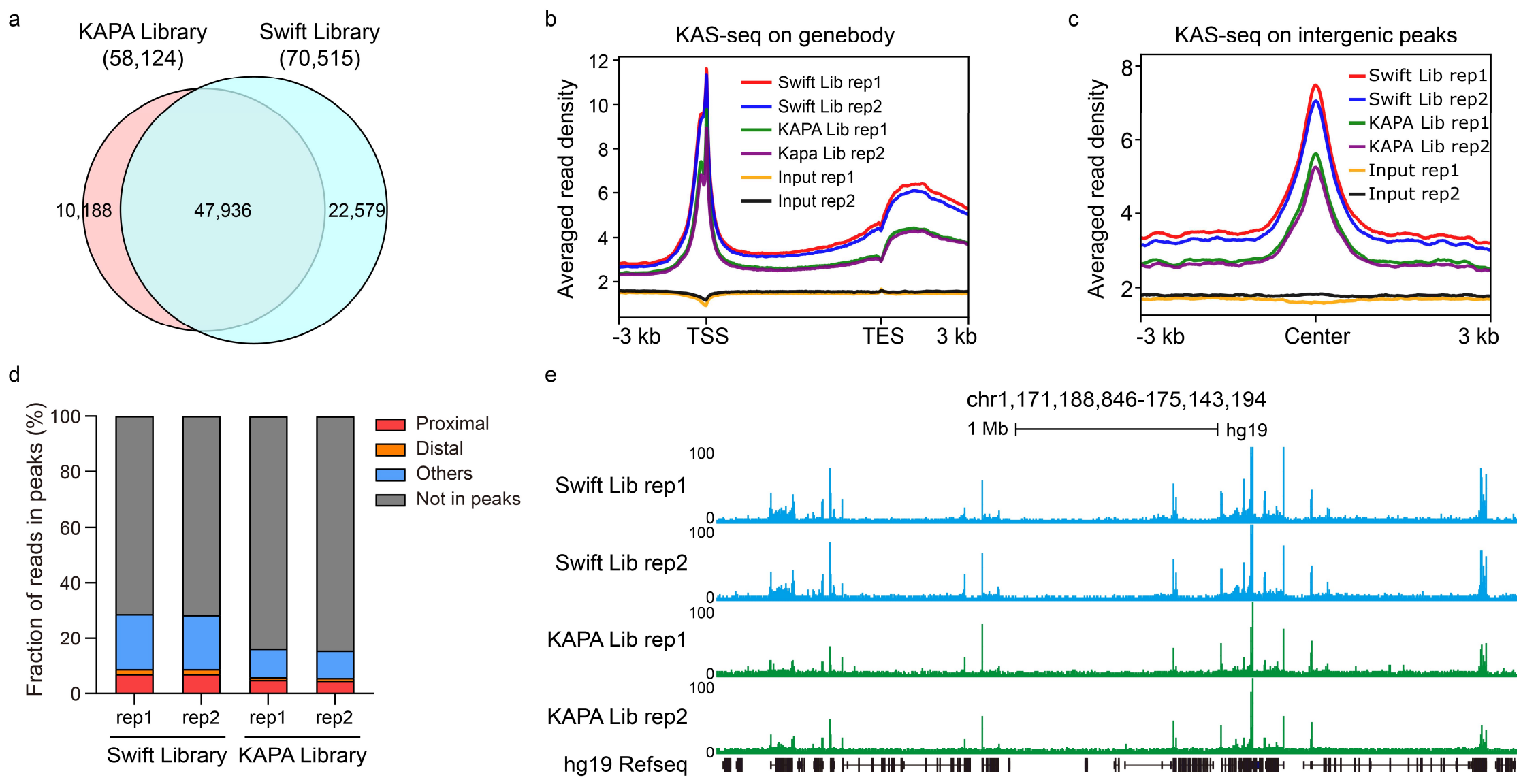

Response Fig. 1: a, Venn diagram showing the overlap of KAS-seq peaks, identified in HEK293T cells using *Opti*-KAS-seq libraries constructed with both Swift Accel-NGS Methyl-seq kit and KAPA HyperPrep kit. **b,** Metagene profile showing the distribution of KAS-seq signals using *Opti*-KAS-seq libraries prepared with Swift Accel-NGS Methyl-seq and KAPA HyperPrep kits at gene-coding regions (n = 36,231) in HEK293T cells, with 3 kb upstream of TSS and 3 kb downstream of TES shown. **c,** Metagene profile showing the distribution of KAS-seq signals using *Opti*-KAS-seq libraries prepared with Swift Accel-NGS Methyl-seq and KAPA HyperPrep kits at intergenic KAS-seq peaks (n = 10,838) in HEK293T cells, with 3 kb upstream of TSS and 3 kb downstream of KAS-seq peaks center shown. **d,** Stacked bar plot showing the fraction of reads in peaks (FRiP), calculated from *Opti*-KAS-seq data prepared using Swift Accel-NGS Methyl-seq and KAPA HyperPrep kits. These KAS-seq reads are uniquely mapped to promoters (±500 bp of TSS), distal cis-regulatory elements (CREs) (>500 bp from TSS) and other regions in HEK293T cells. All values were determined from 30 million randomly selected mapped reads. **e,** Snapshot from UCSC genome browser tracks showing Opti-KAS-seq data, prepared with Swift Accel-NGS Methyl-seq and KAPA HyperPrep kits, across a representative region (chr1:171,188,846-175,143,194). The data indicate that the Swift Accel-NGS Methyl-seq kit offers superior sensitivity, specificity, and overall efficiency in preparing Opti-KAS-seq libraries.

2. Can the authors integrate KAS-ATAC-seq measurements with Pol II pausing index (as measured by either mNET-seq or PRO-seq). Does the extent of ssDNA accumulation track with Pol II pausing?

Response: We appreciate the reviewer's suggestion. KAS-ATAC-seq allows intricate probing of transcriptional activities within CREs by capturing ssDNA at ATAC-seq peaks, which predominantly locate at promoter regions. Meanwhile, *Opti*-KAS-seq

captures ssDNA produced by transcriptionally active RNA polymerases at both promoters and gene body. Because Pol II pausing index is calculated by determining the ratio of read density at the promoter-proximal region (TSS ± 500 bp) to that in the gene body, we hypothesize that *Opti*-KAS-seq is better suited for measuring Pol II pausing index compared to KAS-ATAC-seq.

To confirm this hypothesis, we performed a correlation analysis using *Opti*-KAS-seq, KAS-ATAC-seq, RNA Pol II ChIP-seq, and the pausing index calculated from *Opti*-KAS-seq, PRO-seq, and mNET-seq. Our results indicate that various measurements of the Pol II pausing index, including those derived from *Opti*-KAS-seq, PRO-seq, and mNET-seq, correlate well (Response Fig. 2a). Specifically, the pausing index calculated using Opti-KAS-seq shows a strong correlation with the pausing index calculated using PRO-seq (R = 0.52) (Response Fig. 2b). In contrast, KAS-ATAC-seq exhibits a very low correlation with the pausing index measured by PRO-seq (R = 0.068) and mNET-seq (R = -0.03) (Response Fig. 2c). These results highlight the robustness of *Opti*-KAS-seq in capturing the dynamics of transcriptionally active RNA Pol II.

Additionally, our analysis reveals that both *Opti*-KAS-seq and KAS-ATAC-seq read densities exhibit high correlations with RNA Pol II ChIP-seq (R = 0.78 and R = 0.55, respectively) (Response Fig. 2d-e), suggesting that ssDNA accumulation on promoters detected by these methods is associated with RNA Pol II pausing. Additionally, there is a strong correlation (R = 0.71) between KAS-ATAC-seq and *Opti*-KAS-seq read densities (Response Fig. 2f), indicating that both methods capture similar features of ssDNA accumulation at promoters.

In summary, KAS-ATAC-seq effectively reflects RNA Pol II pausing density at promoters and strongly correlates with *Opti*-KAS-seq measurements. The accumulation of ssDNA at promoters, as detected by both KAS-ATAC-seq and *Opti*-KAS-seq, is indeed associated with RNA Pol II pausing. This underscores the value of KAS-ATAC-seq in analyzing RNA Pol II pausing at transcription initiation sites.

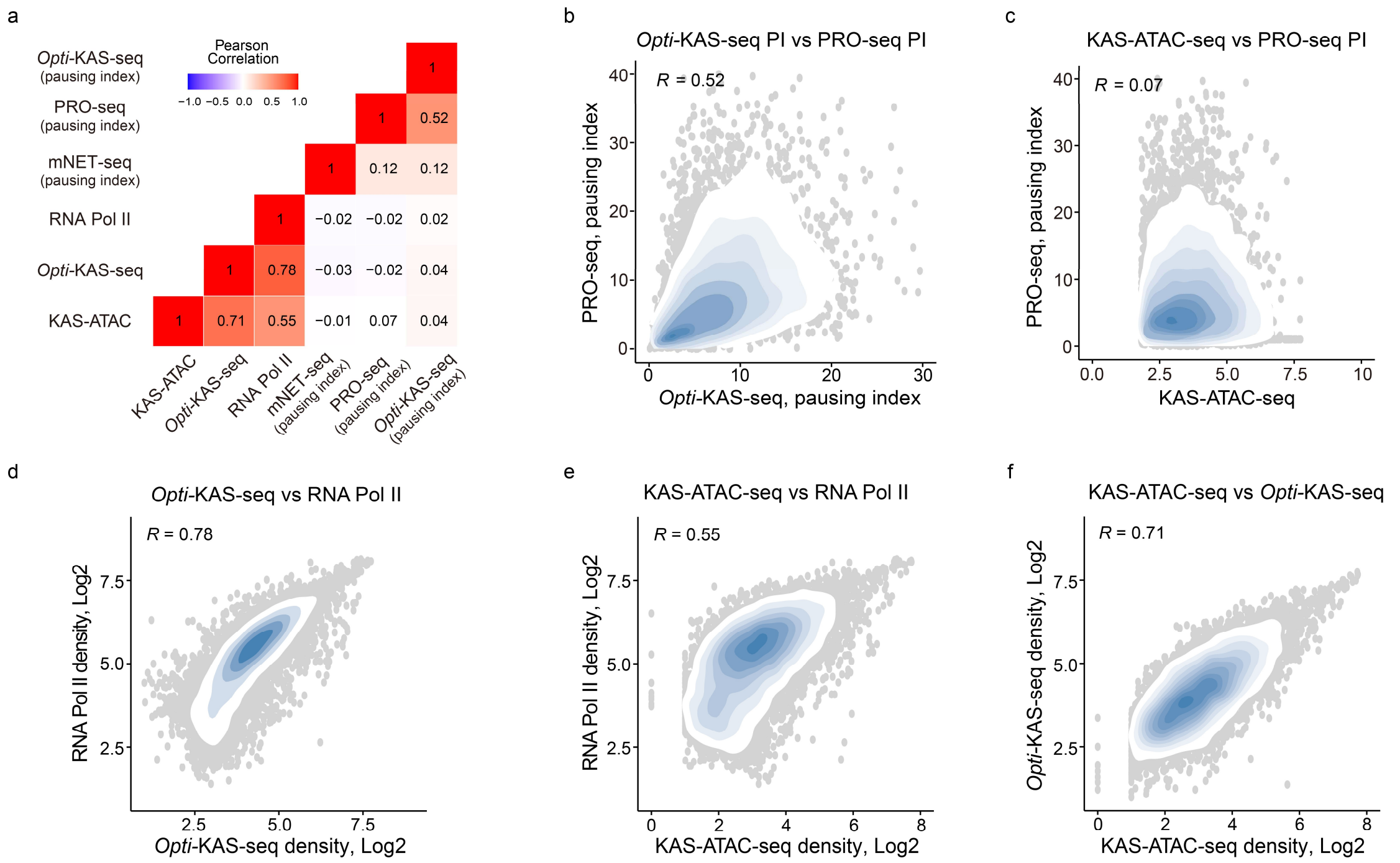

Response Fig. 2: a, Correlation heatmap showing Pearson correlation coefficients among *Opti*-KAS-seq, KAS-ATAC-seq, RNA Pol II ChIP-seq, and pausing index calculated by *Opti*-KAS-seq, PRO-seq, and mNET-seq (n = 9,149). Read density on promoters determines the values for *Opti*-KAS-seq, KAS-ATAC-seq, and RNA Pol II ChIP-seq. **b,** Scatterplot showing the Pearson correlation between pausing index (PI) calculated using *Opti*-KAS-seq and PRO-seq (n = 9,149). The Pearson correlation coefficient (R) is displayed at the top of the plot. Points are color-coded in light blue to indicate SSTE density. **c,** Scatterplot showing the Pearson correlation between pausing index (PI) calculated from PRO-seq and KAS-ATAC-seq read density at promoters (n = 9,149). The Pearson correlation coefficient (R) is displayed at the top of the plot. Points are color-coded in light blue to indicate SSTE density. **d,** Scatterplot showing the Pearson correlation between *Opti*-KAS-seq and RNA Pol II ChIP-seq read density at promoters (n = 9,149). The Pearson correlation coefficient (R) is displayed at the top of the plot. Points are color-coded in light blue to indicate SSTE density. **e,** Scatterplot showing the Pearson correlation between KAS-ATAC-seq and RNA Pol II ChIP-seq read density at promoters (n = 9,149). The Pearson correlation coefficient (R) is displayed at the top of the plot. Points are color-coded in light blue to indicate SSTE density. **e,** Scatterplot showing the Pearson correlation between KAS-ATAC-seq and *Opti*-KAS-seq read density at promoters (n = 9,149). The Pearson correlation coefficient (R) is displayed at the top of the plot. Points are color-coded in light blue to indicate SSTE density.

Reviewer #2:

Comments for the Author:

In this manuscript, Lyu, Gao, and Wu et al. described developing an innovative approach for the analysis of cis-regulatory elements (CREs). The authors first developed an optimized KAS-seq (*Opti*-KAS-seq) with significantly improved performances in analyzing primary cells and tissue samples. Next, they integrated the *Opti*-KAS-seq with ATAC-seq and further developed the KAS-ATAC-seq that identifies the location of CREs and measures their activity states simultaneously. To demonstrate the utilities of the new method, they further applied it to mESCs differentiation into NPCs and uncovered different groups of CREs with distinct regulatory roles during this process. The investigation is comprehensive, the data presentation is clear, and the manuscript is well-written. This method will be a timely contribution to the epigenomics community.

Response: We are grateful for the reviewer's appreciation of this work and constructive comments.

Major points:

1. What is the required sequencing depth for *Opti*-KAS-seq? Could the authors perform a power analysis (i.e., downsample the #s of sequenced reads) for peak calling comparisons between *Opti*-KAS-seq and KAS-seq?

Response: We appreciate the comment regarding the required sequencing depth for *Opti*-KAS-seq. As recommended by the reviewer, we performed a power analysis by down-sampling the number of uniquely mapped reads of KAS-seq and *Opti*-KAS-seq data generated in HEK293T cells. We calculated the percentage of genomic coverage of KAS-seq peaks as a function of sequencing depth, ranging from 0 to 60 million reads.

In HEK293T cells, *Opti*-KAS-seq demonstrates superior performance compared to KAS-seq across all sequencing depths. Specifically, *Opti*-KAS-seq reaches a higher percentage of detected peaks at lower sequencing depths, indicating its higher sensitivity in capturing ssDNA. For example, in HEK293T cells, *Opti*-KAS-seq achieves over 80% peak detection at around 40 million uniquely mapped reads, while KAS-seq requires approximately 50 million uniquely mapped reads to reach a similar level of peak detection (Response Fig. 3).

Based on our power analysis, we conclude that *Opti*-KAS-seq requires a lower sequencing depth than KAS-seq to achieve robust peak calling, emphasizing its advantage in detecting transcriptionally active regions of the genome. To obtain optimal ssDNA enrichment in practical applications of *Opti*-KAS-seq, we recommend a minimum sequencing depth of 40 million uniquely mapped reads. We have included the recommended minimum sequencing depth for *Opti*-KAS-seq in the "Method" session of this manuscript.

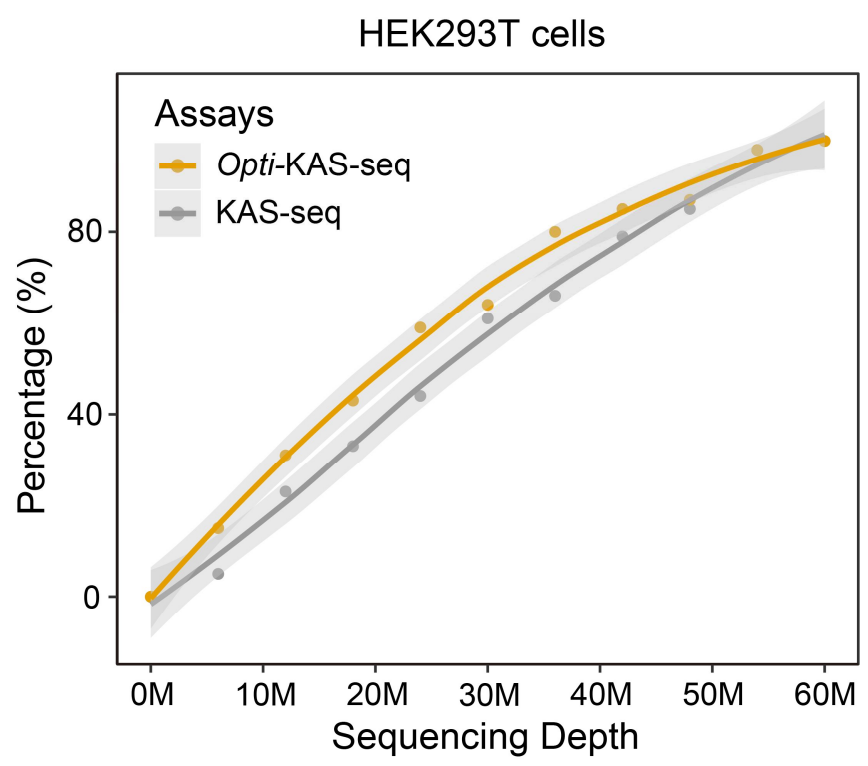

Response Fig. 3: Genomic coverage of detected peaks from *Opti*-KAS-seq and KAS-seq data in HEK293T cells as a function of sequencing depth, ranging from 0 to 60 million reads. *Opti*-KAS-seq demonstrates better performance, achieving over 80% peak detection at approximately 40 million uniquely mapped reads, whereas KAS-seq requires around 50 million uniquely mapped reads to reach a similar level of peak genomic coverage.

2. In Line 190 and Supplementary Fig. 5b, it is a bit unclear what the "correlations" between identified proteins and DOI are. Are they TF binding motifs enrichment scores, genome-wide read distributions/colocalization of protein ChIP-seq reads and DOI, or other measurements?

Response: We apologize for not clearly defining the concept of "correlation" between transcription factors (TFs) and the DNA Openness Index (DOI), as well as the data types used for TF binding detection. The DOI is a metric we specifically designed to evaluate the openness of double-stranded DNA (dsDNA) by calculating the ratio of KAS-ATAC-seq to ATAC-seq signals across both proximal and distal cis-regulatory elements (CREs). This metric provides insights into the transcriptional activity within these regulatory sequences, serving as a quantitative indicator of DNA transcriptional engagement.

We performed Pearson correlation analysis to examine the relationship between DOI, RNA Pol II, and selected TFs binding at proximal CREs. A higher correlation between TF binding and DOI suggests that these TFs are actively involved in promoting RNA Pol II-mediated transcription engagement, indicating their significant regulatory roles compared to other TFs. Additionally, we used ChIP-seq read densities enriched on proximal CREs, derived from TF ChIP-seq data, to calculate the Pearson correlation coefficients.

In summary, the correlations between the identified TF binding affinity on chromatin and DOI reflect the extent to which TFs chromatin binding actively promotes RNA Pol II-mediated transcription, as measured by the DOI. This analysis helps identify TFs that are likely key regulators of transcriptional activity in these proximal CREs. We have revised the manuscript accordingly to clarify the concept of "correlation" between transcription factors (TFs) and the DNA Openness Index (DOI), as well as the data types used to detect TF binding.

3. Lines 273-276, the earlier activated promoter and SSTEs are activated on Days 4 & 6 (for promoters) and Day 4 (for SSTEs). Does this suggest distal enhancers are activated prior to promoters? Or is there also a portion of CREs (but in a lower percentage compared to the promoters C3&4 and SSTEs C4) that are activated earlier in KAS-ATAC-seq?

Response: We appreciate the reviewer's comments regarding the activation timing of promoters and SSTEs during neural differentiation. Arner et al. used cap analysis of gene expression (CAGE) to analyze gene enhancer and promoter activities in various human and mouse cell types[3]. They proposed a general model in which enhancer transcription is the earliest event in successive waves of transcriptional change during cellular differentiation or activation. Conversely, Hirabayashi et al. used native elongating transcript-cap analysis of gene expression (NET-CAGE) and found that transcription of enhancers and promoters were activated simultaneously during cellular stimulation with HRG growth factor in MCF7 cells[4]. They suggested that discrepancies could be due to CAGE being performed on total RNAs, where long-lived mRNAs take longer to accumulate and peak than unstable eRNAs[4].

To explore the activation timing of promoters and SSTEs during mouse neural differentiation induced by retinoic acid (RA) treatment, we identified those that are activated on Days 4 and 6 using KAS-ATAC-seq and ATAC-seq data, respectively. To confirm that these activated promoters and SSTEs function as enhancer-promoter pairs for transcription activation, we included those located within a 100kb range upstream or downstream of each other in our analysis and analyzed their signal dynamics from Day 0 to Day 8. Our findings indicate that promoters and SSTEs were activated simultaneously during mouse neural differentiation induced by RA treatment, as shown by KAS-ATAC-seq data (Response Fig. 4a). This is further supported by ATAC-seq data (Response Fig. 4b). Specifically, in the genes Gli2 and Ier2, both KAS-ATAC-seq and ATAC-seq data show simultaneous activation of promoters and SSTEs at Day 4 (Response Fig. 4c).

Additionally, we indeed observed that a portion of promoters and SSTEs were activated earlier in KAS-ATAC-seq than ATAC-seq (Response Fig. 4a-c; Fig. 5d-g and Extended Data Fig. 7i-j). This is likely because KAS-ATAC-seq signals reflect real-time transcription levels by detecting ssDNA, whereas ATAC-seq signals indicate chromatin accessibility on CREs. The faster of transcriptional activation of CREs and corresponding genes, the greater the time lag between KAS-ATAC-seq and ATAC-seq peaks.

Taken together, our findings suggest that promoters and SSTEs are typically activated simultaneously during neural differentiation induced by RA treatment. Additionally, we observed that some transcriptionally activated promoters and SSTEs are activated earlier in KAS-ATAC-seq than ATAC-seq. The analysis of promoters and SSTEs activation timing during neural differentiation has been included as Supplementary Fig. 8 in the manuscript. We also have discussed the possible reasons for the earlier activation of certain CREs in KAS-ATAC-seq compared to ATAC-seq in the "Discussion" section of this manuscript.

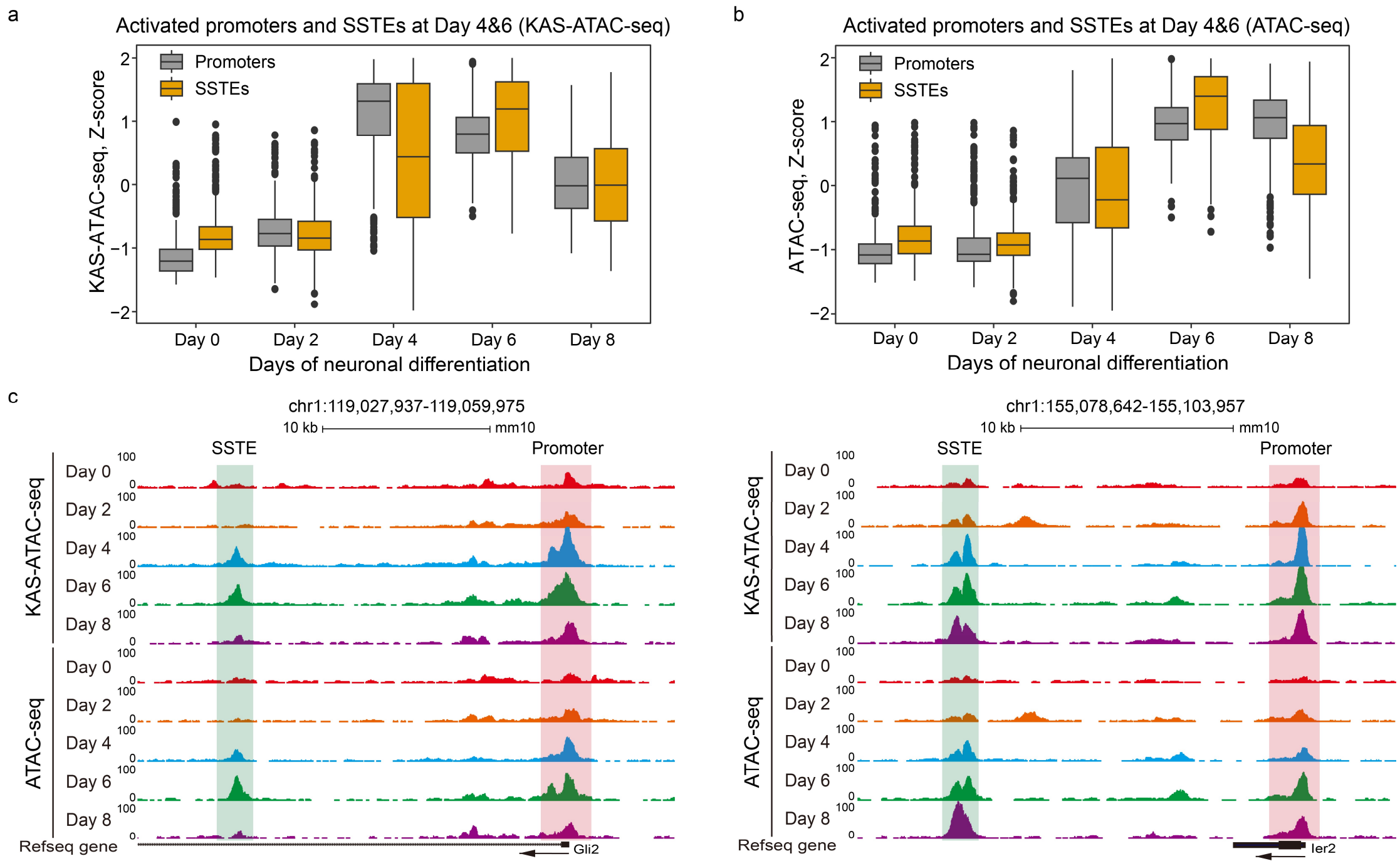

Response Fig. 4: a, Grouped boxplot showing z-scores of activated promoters (grey boxes) and SSTEs (yellow boxes) identified on Days 4 and 6 of neural differentiation after RA treatment. Z-scores were calculated using KAS-ATAC-seq data from Day 0 and 8 during neural differentiation of mESCs to NPCs. **b,** Grouped boxplot showing z-scores of activated promoters (grey boxes) and SSTEs (yellow boxes) identified on Days 4 and 6 of neural differentiation after RA treatment. Z-scores were calculated using ATAC-seq data from Days 0 and 8 during neural differentiation of mESCs to NPCs. **c,** Snapshots of UCSC genome browser tracks showing ATAC-seq and KAS-ATAC-seq data on the promoters and SSTEs of two example genes, *Gli2* (left panel) and *Ler2* (right panel), at specific time point from Day 0 to Day 8 during mouse neural differentiation from mESCs to NPCs.

4. The different roles of SSTEs and DSEs in TAD boundaries are exciting. Could the author discuss the feasibility of using KAS-ATAC-seq & ATAC-seq data to identify CREs & TAD boundaries?

Response: We appreciate the reviewer's interest in exploring the distinct roles of Single-Stranded Transcribing Enhancers (SSTEs) and Double-Stranded Elements (DSEs) at topologically associating domain (TAD) boundaries. We agree with the reviewer that different types of CREs defined using KAS-ATAC-seq and ATAC-seq data could significantly improve the identification of TAD boundaries, presenting a promising and feasible approach. ATAC-seq provides a genome-wide profile of chromatin accessibility, allowing the detection of open chromatin regions where transcription factors and other regulatory proteins bind. KAS-ATAC-seq, by capturing ssDNA associated with active transcription, offers additional insights at these open chromatin sites.

In this manuscript, we found that SSTEs, particularly stable SSTEs (S-SSTEs), are predominantly localized within TADs, while DSEs are strongly localized towards TAD boundaries. Our analysis further revealed that SSTEs and promoters are closely

associated with RNA Pol II- and YY1-mediated long-range interactions. In contrast, DSEs primarily align with CTCF- and Cohesin-mediated long-range interactions typically found in insulator regions that delineate TAD boundaries. These findings support the observed enrichment preferences of SSTEs within TADs and DSEs at TAD boundaries. While KAS-ATAC-seq and ATAC-seq provide valuable insights into defining SSTEs and DSEs, identifying TAD boundaries still largely requires chromatin conformation capture techniques such as Hi-C, which directly show physical genome interactions. Integrating KAS-ATAC-seq and ATAC-seq data with Hi-C data can help eliminate false positives and enhance the precise identification of TAD boundaries. Our study found that open chromatin regions without transcription (DSEs), identified by ATAC-seq and KAS-ATAC-seq, often coincide with TAD boundaries, as these regions are frequently occupied by CTCF and the Cohesin protein complex.

In conclusion, we believe that employing KAS-ATAC-seq and ATAC-seq datasets to identify TAD boundaries is both feasible and effective. However, for precise delineation of TAD boundaries, it is essential to integrate these datasets with chromatin interaction data from techniques such as Hi-C. This integration not only improves the resolution and accuracy in pinpointing CREs and TAD boundaries but also enriches our understanding of genomic architecture and its impact on transcriptional regulation. The feasibility of using KAS-ATAC-seq and ATAC-seq data to identify TAD boundaries has been further discussed in the "Discussion" section of this manuscript.

References:

1. Schumacher, C., Kurihara, L. & Cunningham, K. (Nature Publishing Group US New York, 2015).
2. Van Kets, V. *et al.* Kapa Hyper Prep: A next-generation kit for fast and efficient library construction from challenging DNA samples. *Adv Genome Biol Technol* (2014).
3. Arner, E. *et al.* Transcribed enhancers lead waves of coordinated transcription in transitioning mammalian cells. *Science* **347**, 1010-1014 (2015).
4. Hirabayashi, S. *et al.* NET-CAGE characterizes the dynamics and topology of human transcribed cis-regulatory elements. *Nature genetics* **51**, 1369-1379 (2019).

REVIEWERS' COMMENTS

Reviewer #1 (Remarks to the Author):

The authors have addressed all of my comments.

Reviewer #2 (Remarks to the Author):

The authors have fully addressed all my concerns.
