# Supplementary material for: Quantitative analysis of cis-regulatory elements in transcription with KAS-ATAC-seq
Source: Nat Commun. 2024 Aug 10;15:6852. doi: 10.1038/s41467-024-50680-8 (PMC11316786; doi:10.1038/s41467-024-50680-8)
Supplement: Supplementary file 3 [file 41467_2024_50680_MOESM6_ESM.pdf]

nature portfolio

Corresponding author(s): Chuan He

Last updated by author(s): Jun 21, 2024

Reporting Summary

Nature Portfolio wishes to improve the reproducibility of the work that we publish. This form provides structure for consistency and transparency in reporting. For further information on Nature Portfolio policies, see our Editorial Policies and the Editorial Policy Checklist.

Statistics

For all statistical analyses, confirm that the following items are present in the figure legend, table legend, main text, or Methods section.

n/a	Confirmed	
☐	☒	The exact sample size (*n*) for each experimental group/condition, given as a discrete number and unit of measurement
☐	☒	A statement on whether measurements were taken from distinct samples or whether the same sample was measured repeatedly
☐	☒	The statistical test(s) used AND whether they are one- or two-sided<br>*Only common tests should be described solely by name; describe more complex techniques in the Methods section.*
☒	☐	A description of all covariates tested
☒	☐	A description of any assumptions or corrections, such as tests of normality and adjustment for multiple comparisons
☐	☒	A full description of the statistical parameters including central tendency (e.g. means) or other basic estimates (e.g. regression coefficient) AND variation (e.g. standard deviation) or associated estimates of uncertainty (e.g. confidence intervals)
☐	☒	For null hypothesis testing, the test statistic (e.g. *F*, *t*, *r*) with confidence intervals, effect sizes, degrees of freedom and *P* value noted<br>*Give P values as exact values whenever suitable.*
☒	☐	For Bayesian analysis, information on the choice of priors and Markov chain Monte Carlo settings
☒	☐	For hierarchical and complex designs, identification of the appropriate level for tests and full reporting of outcomes
☐	☒	Estimates of effect sizes (e.g. Cohen's *d*, Pearson's *r*), indicating how they were calculated

Our web collection on statistics for biologists contains articles on many of the points above.

Software and code

Policy information about availability of computer code

Data collection	Illuminal NextSeq 500, Illumina NovaSeq 6000, Illuminal MiSeq.
Data analysis	Cutadapt (v4.2), Bowtie2 (v2.4.4), STAR (v2.7.9a), Hisat2(v2.2.1), RNASeQC (v2.4.2), featureCounts (v2.0.3), deepTools (v3.5.1), HOMER (v4.11), MACS2(v2.2.7.1), epic2(0.0.52), UCSC Genome Browse, Genomic Regions Enrichment of Annotations Tool (GREAT, v4.0.4), Samtools (v1.16.1), BEDTools (v2.30.0), Juicer(v1.6), R (v3.6.3), corrplot (v0.92) R package, ATACseqQC (v1.28.0) R package, clusterProfiler (v4.4.4), GraphPad Prism 7.<br>Codes for processing KAS-seq and KAS-ATAC-seq data are available in the following GitHub repository: https://github.com/Ruitulyu/KAS-Analyzer.

For manuscripts utilizing custom algorithms or software that are central to the research but not yet described in published literature, software must be made available to editors and reviewers. We strongly encourage code deposition in a community repository (e.g. GitHub). See the Nature Portfolio guidelines for submitting code & software for further information.

Data

Policy information about availability of data

All manuscripts must include a data availability statement. This statement should provide the following information, where applicable:

- Accession codes, unique identifiers, or web links for publicly available datasets
- A description of any restrictions on data availability
- For clinical datasets or third party data, please ensure that the statement adheres to our policy

Data availability
The raw and processed data of Opti-KAS-seq, KAS-seq, KAS-ATAC-seq, and ATAC-seq experiments performed using HEK293T cells and mouse embryonic stem cells have been deposited in the Gene Expression Omnibus (https://www.ncbi.nlm.nih.gov/geo/) under the accession number: GSE256232 [https://www.ncbi.nlm.nih.gov/geo/query/acc.cgi?acc=GSE256232]. All published datasets reanalyzed in this study were summarized in Supplementary Table 3. The raw data generated in this study are provided in the Source Data file.

Research involving human participants, their data, or biological material

Policy information about studies with human participants or human data. See also policy information about sex, gender (identity/presentation), and sexual orientation and race, ethnicity and racism.

Reporting on sex and gender	This work does not involve human research participants.
Reporting on race, ethnicity, or other socially relevant groupings	This work does not involve human research participants.
Population characteristics	This work does not involve human research participants.
Recruitment	This work does not involve human research participants.
Ethics oversight	This work does not involve human research participants.

Note that full information on the approval of the study protocol must also be provided in the manuscript.

Field-specific reporting

Please select the one below that is the best fit for your research. If you are not sure, read the appropriate sections before making your selection.

☒ Life sciences ☐ Behavioural & social sciences ☐ Ecological, evolutionary & environmental sciences

For a reference copy of the document with all sections, see nature.com/documents/nr-reporting-summary-flat.pdf

Life sciences study design

All studies must disclose on these points even when the disclosure is negative.

Sample size	No statistical methods were used to predetermine sample size. Instead, sample sizes were chosen based on standard practices and previous literature within our field, which have historically provided sufficient power to detect meaningful effects and differences in similar studies.
Data exclusions	The adapter and low-quality sequences were trimmed from the raw sequencing data. When comparing KAS-ATAC-seq with nascent RNA-seq, CREs on the gene body were excluded to avoid potential transcription signals related to elongation in nascent RNA-seq data.
Replication	All KAS-seq, Opti-KAS-seq, ATAC-seq, and KAS-ATAC-seq experiments in this study were conducted with at least two biological replicates, unless specified otherwise. We performed Pearson correlation analysis and can confirm that the data were significantly reproducible.
Randomization	The experiments were not randomized. Controlling for covariates was unnecessary because all assays were performed in pairs.
Blinding	Blinding was not applicable because the focus of this paper is the development of a new method to provide quantitative insights into transcriptional activity of CREs, and did not involve group allocation and blinding.

Reporting for specific materials, systems and methods

We require information from authors about some types of materials, experimental systems and methods used in many studies. Here, indicate whether each material, system or method listed is relevant to your study. If you are not sure if a list item applies to your research, read the appropriate section before selecting a response.

Materials & experimental systems

n/a	Involved in the study
☒	☐ Antibodies
☐	☒ Eukaryotic cell lines
☒	☐ Palaeontology and archaeology
☐	☒ Animals and other organisms
☒	☐ Clinical data
☒	☐ Dual use research of concern
☒	☐ Plants

Methods

n/a	Involved in the study
☒	☐ ChIP-seq
☐	☒ Flow cytometry
☒	☐ MRI-based neuroimaging

Eukaryotic cell lines

Policy information about cell lines and Sex and Gender in Research

Cell line source(s)	HEK293T cells were purchased from ATCC (CRL11268).<br>Murine embryonic stem (ES) cells were purchased from ATCC (CRL-1821).
Authentication	Cell lines were not authenticated after purchase from ATCC.
Mycoplasma contamination	All cell lines used in this study were tested negative of mycoplasma contamination.
Commonly misidentified lines<br>(See ICLAC register)	No commonly misidentified line was used.

Animals and other research organisms

Policy information about studies involving animals; ARRIVE guidelines recommended for reporting animal research, and Sex and Gender in Research

Laboratory animals	This study involve laboratory mouse. Male B6 mice were purchase from the Jackson Laboratory (catalog no. C57BL/6J). All mice were used at 6-12 weeks of age. Mice were housed under pathogen-free conditions per the National Institutes of Health (NIH) Guide for the Care and Use of Laboratory Animals. Mice were maintained a 12-hour light/12-hour dark cycle, with an ambient temperature of 22°C (±2°C) and relative humidity of 50-60%. These conditions were carefully monitored to ensure a consistent and suitable environment for the animals throughout the duration of the study. All animal care and experiments were approved by the University of Chicago Institutional Animal Care and Use Committee (IACUC), and are compliant with all relevant ethical regulations regarding animal research.
Wild animals	This study did not involve wild animals.
Reporting on sex	This study did not involve sex.
Field-collected samples	This study did not involve samples collected from field.
Ethics oversight	Mice were housed under pathogen-free conditions per the National Institutes of Health (NIH) Guide for the Care and Use of Laboratory Animals. All animal care and experiments were approved by the University of Chicago Institutional Animal Care and Use Committee (IACUC), and are compliant with all relevant ethical regulations regarding animal research.

Note that full information on the approval of the study protocol must also be provided in the manuscript.

Plants

Seed stocks	This study did not involve seed stocks.
Novel plant genotypes	This study did not involve novel plant.
Authentication	This study did not involve seed stocks and novel plant.

Flow Cytometry

Plots

Confirm that:

☒ The axis labels state the marker and fluorochrome used (e.g. CD4-FITC).

☒ The axis scales are clearly visible. Include numbers along axes only for bottom left plot of group (a 'group' is an analysis of identical markers).

☒ All plots are contour plots with outliers or pseudocolor plots.

☒ A numerical value for number of cells or percentage (with statistics) is provided.

Methodology

Sample preparation	To prepare HEK293T cells for FACS analysis using Hoechst 33342 staining, begin by culturing the cells in DMEM supplemented with 10% FBS and 1% penicillin-streptomycin at 37°C in a 5% CO2 atmosphere. Harvest the cells at 70-80% confluency using trypsin-EDTA, neutralize with medium containing FBS, and centrifuge at 300 x g for 5 minutes. Resuspend the cell pellet in PBS and adjust the concentration to 1x10^6 cells/mL. For staining, add Hoechst 33342 directly to the cell suspension, typically at a final concentration of 1-10 µg/mL, and incubate for 15-30 minutes at 37°C in the dark. Post-staining, wash the cells by adding cold PBS and centrifuge to remove excess dye. Resuspend in FACS buffer and filter through a 35 µm strainer to ensure a single-cell suspension. Keep cells on ice until FACS analysis to maintain viability and prevent dye efflux.
Instrument	BD FACSAriaIIIu
Software	FACSDiva Version 6.1.3
Cell population abundance	G1=26.4%, S=18.5
Gating strategy	We established a gate around the main population cluster visible in a FSC vs. SSC to exclude debris, dead cells, and aggregates.

☒ Tick this box to confirm that a figure exemplifying the gating strategy is provided in the Supplementary Information.
